# Counterion-Free Ionic Associating Polymers: *In Situ* Ionization and Coupling of Alkyl Sulfonate Precursors

**DOI:** 10.1021/acs.macromol.5c01487

**Published:** 2025-08-25

**Authors:** Jie Xu, Chia-Chi Tsai, Oscar Nordness, Shuyi Xie

**Affiliations:** † Department of Chemical Engineering, 14736 Texas A&M University, College Station, Texas 77843, United States; ‡ Henry Krumb School of Mines, Earth and Environmental Engineering Department, 5798 Columbia University, New York, New York 10027, United States

## Abstract

Mixing oppositely charged cationic and anionic polymer salts (poly^+^X^–^ and poly^–^Y^+^) typically yields ionic associating polymers (IAPs) coexisting with counterions (X^–^/Y^+^). These counterions screen interchain Coulombic interactions and weaken polymer association. Herein, we present an innovative and straightforward strategy to synthesize counterion-free IAPs based on two charge-neutral telechelic oligomers A2 and B2, bearing imidazole and ethyl sulfonate end groups, respectively. Notably, we have developed a novel base-free salt metathesis route to synthesize B2 with nearly quantitative chain-end fidelity (>97%). It successfully overcame issues of unstable intermediates and basic conditions encountered in the conventional route. Reactive melt blending of A2 and B2 results in *in situ* ionization and chain coupling, producing a polymer melt characterized by a 2-fold increase in viscosity due to aprotic and reversible ionic associations. The viscosity and self-diffusion of the IAP were quantified by rheology and pulsed-field gradient nuclear magnetic resonance (PFG-NMR) spectroscopy, respectively. Notably, the product of diffusion coefficient and viscosity (*D*η) positively deviates from the Rouse model prediction, consistent with the formation of a transient dynamic network in which chain mobility is partially decoupled from macroscopic viscosity. We anticipate that this modular synthesis approach can be readily extended to other synthetic polymer systems, where the strength of ionic interactions can be systematically tuned. Such control would guide the design of dynamic polymeric materials that assemble and disassemble on demand, offering enhanced recyclability and sustainability.

## Graphical abstract

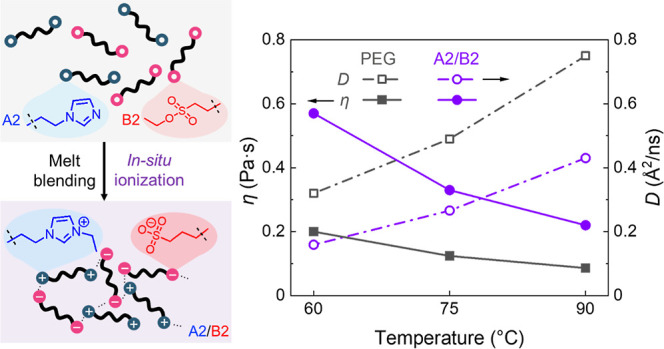

## Introduction

Coulombic interactions between oppositely charged ions can be leveraged to construct materials with precisely defined structures and properties. Examples include ionic crystals (e.g., table salt), where compact ions are linked by strong ionic bonds to form long-range ordered lattice. As ions get more diffuse, lattice energy decreases, leading to less ordered materials that shift from crystalline to liquid crystalline or even amorphous states.^1,2^ For instance, bulky ion pairs form ionic liquids (ILs) with melting points below 100 °C. While ILs (or ionic supramolecules) based on small molecule or surfactant building blocks have been extensively studied, the assembly and dynamics of poly­(ionic liquids)^3–5^ and related ionic associating polymers (IAPs) remain largely unexplored. The ionic bond strength in polymer melts ranges from 1 to 100 *k*_B_ *T*, depending on ion compactness and the dielectric constant of the polymer matrix.^6^ Owing to the excellent bond energy tunability and the vast design space of polymer building blocks, we envision that rationally designed IAPs can yield polymeric materials with more dynamic features. These materials can be assembled and disassembled on demand, offering greater recyclability than their covalently bonded counterparts. Furthermore, the potential for ion transport may render IAPs promising candidates as electrolytes for energy storage applications.

However, conventional blends of a polycation (poly^+^X^–^) and polyanion (poly^–^Y^+^) form a polymer complex (poly^+^/poly^–^) with a certain amount of X^–^Y^+^ counterions that may substantially screen ionic interactions,^7^ thus changing the physiochemical properties of the material (Scheme 1a).^8,9^ Similar to the synthesis of ILs, the removal of counterions from IAPs is challenging, laborious, and economically unfavorable (e.g., extensive washing and dialysis are typically required).^10^ Therefore, it is highly desirable to design and construct counterion-free IAPs to circumvent these arduous purification procedures. To date, the most common approach for synthesizing counterion-free IAPs is to react a polymeric acid with a polymeric base. Proton transfer occurs between the acid and base in this reaction, resulting in the conjugate acid of the base (a protonated cation) and the conjugate base of the acid (an anion).^11–13^ However, acid/base interactions are not purely ionic in nature but are better described as ionic hydrogen bonding.^14^ Such protic systems are also less electrochemically stable due to the labile proton on the cation.^15^ In fact, when proton transfer is incomplete, the proton may be shared between the acid and base, thereby reducing the effective charge (Scheme 1b).^16–18^ At elevated temperatures, such protic IAPs may lose ionic character due to reverse proton transfer.^11,12^

**Scheme 1:**
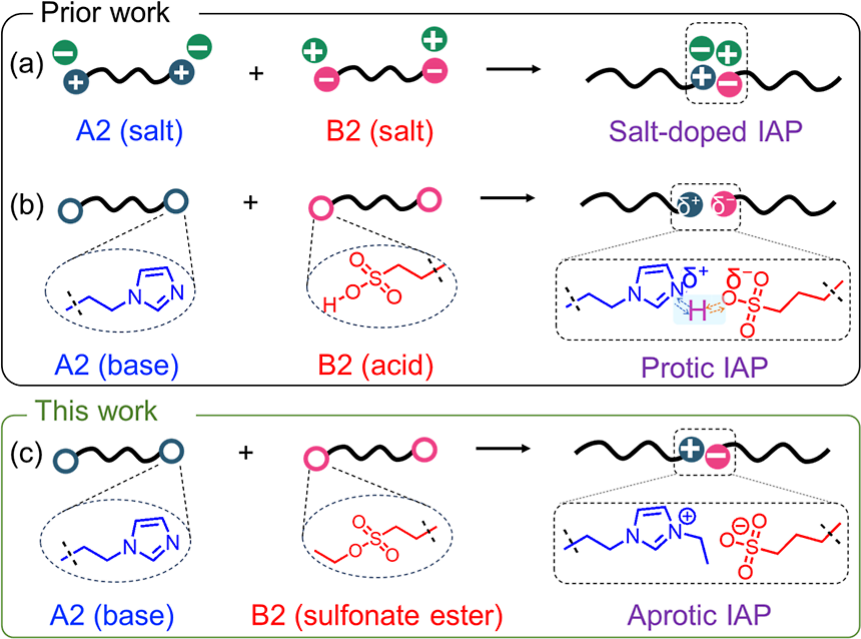
(a) Blending Oppositely Charged Polymers Results in IAPs with Intrinsic Counterions; (b) Blending Acidic and Basic Polymers Results in Counterion-Free IAPs with Protic Ionic Junctions; (c) New Synthesis Route of Counterion-Free Aprotic IAPs via *In Situ* Ionization and Coupling of Sulfonate-Alkyl Precursors

Therefore, an alternative synthetic strategy is desired to create counterion-free IAPs that feature controllable ionic linkages. Such a strategy would address the challenges of tedious purification in counterion-containing blends and the inherent instability of protic systems. Here we report a unique *in situ* ionization strategy to construct counterion-free poly^+^/poly^–^ IAPs based on charge-neutral telechelic oligomers A2 and B2, where A2 is a base (e.g., imidazole) and B2 is a strong alkylating agent (e.g., sulfonate ester). Upon blending, the imidazole end group is quaternized into an alkyl imidazolium cation, while a sulfonate anion is formed simultaneously (Scheme 1c). Notably, the “alkyl chain transfer” from the sulfonate ester to the base is completely irreversible, rendering the resulting ions permanent. Even at an elevated temperature, the poly^+^/poly^–^ system thus still retains its ionic nature. While this *in situ* ionization strategy has previously been applied to synthesize halide-free ILs with high yield and conversion, it has yet to be applied to polymers or polymeric ILs,^19^ potentially due to two main challenges: (1) functionalizing both polymer chain ends with sulfonate esters while maintaining high end-group fidelity and minimizing hydrolysis is difficult; (2) achieving high *in situ* ionization conversion is hindered by the limited diffusion of polymer chains, especially in the melt state. To tackle these two problems, we have developed a new synthetic route for telechelic polymer sulfonate esters under mild reaction conditions, minimizing hydrolysis and achieving high chain-end fidelity. We also selected a flexible polyethylene glycol (PEG) model system to demonstrate high ionization conversion upon melt blending without the use of solvents.

To the best of our knowledge, this is the first synthesis of counterion-free aprotic IAPs. This approach enables near-quantitative functionality and near-complete ionization conversion. We confirmed these results through proton nuclear magnetic resonance (^1^H NMR) spectroscopy and matrix-assisted laser desorption/ionization time-of-flight (MALDI-TOF) mass spectroscopy. The dynamics and structure of the resulting counterion-free IAPs were quantified by small-amplitude oscillatory shear rheology (SAOS), pulsed-field gradient nuclear magnetic resonance (PFG-NMR) spectroscopy, and small- and wide-angle X-ray scattering (SAXS/WAXS). The ionic functionalities are compatible with the PEG backbone without microphase separation. Furthermore, the interchain Coulombic attractions enhance viscosity and induce unique dynamics that deviate from simple Rouse behavior.

## Experimental Section

### Materials

All reagents were purchased from commercial suppliers and used without further purification unless stated otherwise. Poly­(ethylene glycol) (diol PEG) was obtained from Tokyo Chemical Industry and dried under vacuum at 70 °C for several hours prior to use. Triethylamine (TEA, ≥99.5%), methanesulfonyl chloride (MsCl, ≥99.7%), sodium hydride (NaH, 60% dispersion in mineral oil), imidazole (≥99%), 1,3-propane sultone (≥98%), 2-propanol (IPA, anhydrous, ≥99.5%), oxalyl chloride ((COCl)_2_, ≥99%), ethanol (EtOH, anhydrous, ≥99.5%), silver nitrate (AgNO_3_, ≥99.0%), and iodoethane (EtI with copper as the stabilizer, ≥99%) were acquired from Sigma-Aldrich. 1-Ethyl-3-methylimidazolium methanesulfonate ([emim^+^]­[MeSO_3_^–^], ≥98.0%) was purchased from Tokyo Chemical Industry. Solvents, including dichloromethane (anhydrous with 40–150 ppm amylene as the stabilizer, ≥99.8%), water (HPLC grade), tetrahydrofuran (THF, anhydrous, ≥99.9%), methanol (MeOH, anhydrous, ≥99.9%), *N*,*N*-dimethylformamide (anhydrous, 99.8%), and acetonitrile (ACN, ≥99.9%) were obtained from Sigma-Aldrich. Diethyl ether (anhydrous, ≥99.9%) and chloroform-*d* (≥99.8 atom % D) were obtained from Fisher Scientific. Ultrahigh purity nitrogen was obtained from Airgas.

### Methods and Characterization Techniques

^1^H NMR spectroscopy was performed on a Bruker AVANCE Neo 400 Hz spectrometer at 25 °C using chloroform-*d* as the NMR solvent. FTIR measurements were taken on a Nicolet iS5 FTIR with an iD7 ATR accessory. MALDI-TOF mass spectrometry, utilizing DCTB matrix and NaTFA cationization, was employed to analyze polymer molecular weight characteristics (*M*_n_, *M*_w_, *D̵*), summarized in Table S1. Rheological measurements were performed on a TA Instruments AR-G2 rheometer with a parallel-plate geometry under N_2_ purge, including steady shear experiments at 60 °C, temperature sweeps, and dynamic shear experiments within the linear viscoelastic region. SAXS/WAXS patterns were acquired at both synchrotron- and lab-based facilities, with 2D isotropic scattering patterns reduced to 1D scattering intensity as a function of the wavevector *q*. PFG-NMR measurements were performed on a 400 MHz Bruker spectrometer at 60 °C, 75 °C, and 90 °C using a stimulated-echo sequence to determine proton (^1^H) self-diffusion coefficients. Melting temperature (*T*_m_) was determined by a TA Instruments DSC 2500 or Q200, employing heating/cooling cycles with specific ramp rates. Thermal stability was analyzed using a TA Instrument TGA 5500 under N_2_ flow to determine the decomposition temperature (*T*_d_ at 5% weight loss^20^). Polymer molecular weight characteristics (*M*_n_, *M*_w_, *D̵*) were also characterized by a size exclusion chromatograph coupled with multiangle light scattering (SEC-MALS) using THF. Characterization details can be found in the Supporting Information.

## Results and Discussion

### Polymer Synthesis and Characterization

To synthesize the IAPs, we selected an unentangled PEG diol (*M*_n_ = 2800 g/mol, *D̵* ≈ 1.03) as the building block precursor. Following established procedures,^21–24^ reacting PEG diol with methane sulfonyl chloride followed by imidazole yielded A2 (i.e., imidazole-end-capped PEG) with high chain-end fidelity (Scheme 2a). Although a handful of studies have shown that methacrylate and styrene monomers bearing sulfonate anions can be functionalized into sulfonate ester,^25–29^ to the best of our knowledge, the synthesis of a polymer with alkyl sulfonate chain ends (B2) has not been reported. We synthesized the B2 precursor (i.e., sodium sulfonate-end-capped PEG) via ring-opening of 3-propane sultone (Scheme 2b).^30,31^ Following the conventional sulfonate ester synthesis route, the B2 precursor was activated using oxalyl chloride, resulting in a sulfonyl chloride intermediate. Esterification was then performed by adding an ethanol/triethylamine solution (Scheme 2c). This step is essentially a nucleophilic substitution, where ethanol (a nucleophile) attacks the sulfonyl chloride. Since the reaction produces HCl, a base (typically TEA) is required to scavenge the HCl and drive the reaction to completion. However, despite careful optimization of both reaction conditions and purification methods, we were unable to achieve ethyl sulfonate functionality above 80% due to two key challenges: (1) the sulfonyl chloride intermediate is unstable and prone to hydrolysis back to sulfonic acid or sulfonate salt;^27,32^ (2) the final product B2 (ethyl sulfonate end-groups) is extremely sensitive to basic conditions.^26,33^

**Scheme 2:**
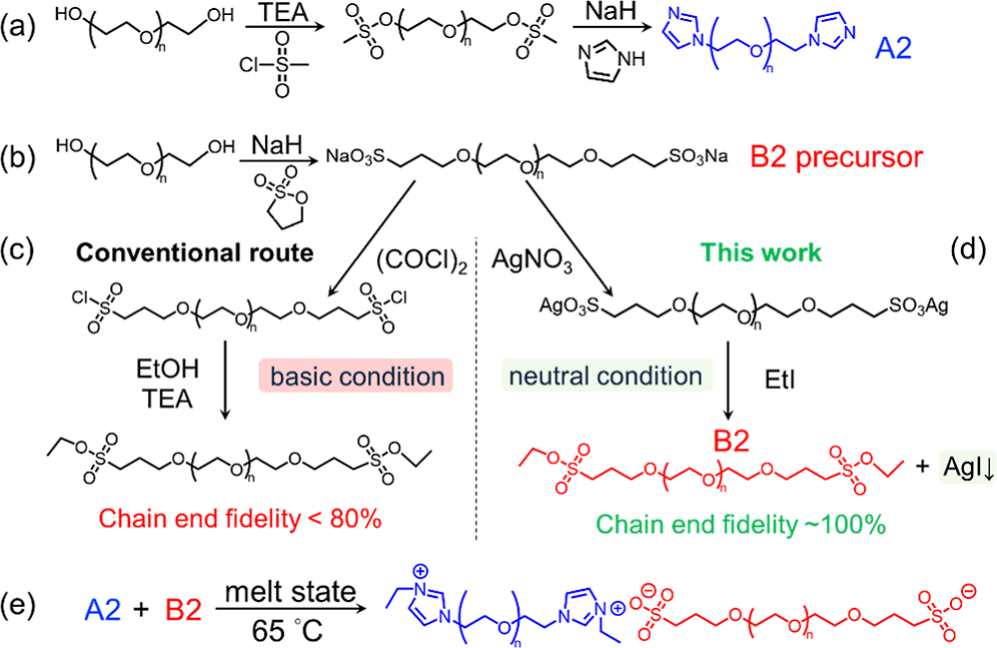
Synthesis Route of (a) A2 (α,ω-Imidazole PEG) and (b) B2 Precursor (α,ω-Sodium Sulfonate PEG); Comparison of Routes to B2 (α,ω-Ethyl Sulfonate PEG): (c) Conventional Route via the Unstable Sulfonyl Chloride Intermediate under Basic Conditions (Low Chain-End Fidelity); (d) New Route via Base-Free Salt Metathesis Reaction (High Chain-End Fidelity); (e) *In Situ* Ionization of Neutral Telechelic Oligomers Resulting in Counterion-Free IAPs

Therefore, to overcome the limitations of the base-catalyzed sulfonyl chloride route, we instead employed a salt metathesis nucleophilic substitution reaction to synthesize α,ω-ethyl sulfonate PEG, B2 (Scheme 2d). In short, the B2 precursor was ion-exchanged into the silver sulfonate form, which was then reacted with ethyl iodide to produce the ethyl sulfonate end group and a salt precipitate (silver iodide). In essence, this reaction is analogous to a Williamson ether synthesis. It is driven by the low solubility of silver iodide, and the pH of the reaction medium remains almost neutral throughout.^34^ This synthetic approach enabled us to achieve nearly quantitative chain-end fidelity in B2 (>97%), enabling well-defined IAP formation via melt state blending. Characteristics for telechelic oligomers and the A2/B2 IAP blend are summarized in Table 1.

**Table 1:** Characteristics for Telechelic Oligomers and the Counterion-Free IAPs

component	sample ID	*M*_n_ (kg/mol)^a^	*D̵* = *M*_w_/*M*_n_ ^b^	*T*_m_ (°C)^c^	*T*_d_ (°C)^d^
α,ω-diol PEG	PEG	2.8	1.03*, 1.01**	60.0	303
α,ω-imidazole PEG	A2	2.9	1.01**	47.9	335
α,ω-ethyl sulfonate PEG	B2	3.1	1.01**	45.8	278
ionic PEG blend	A2/B2 (blend)			47.4	471
IL doped PEG diol	IL/PEG	2.8	1.03*, 1.01**	50.4/55.9	

a Determined via ^1^H NMR spectroscopy.

b Determined via SEC-MALS* or MALDI-TOF** mass spectrometry.

c Determined via DSC.

d Determined via TGA.

We confirmed the successful synthesis of telechelic oligomers (A2 and B2) with near-quantitative chain-end fidelity (>97%) by ^1^H NMR spectroscopy (Figure 1). This is evidenced by the complete disappearance of the terminal diol signal (peak b) and the appearance of characteristic end-group signals (peaks d1 and d2 for A2, and peaks g and h for B2) in the proton spectra. Subsequent solvent-free blending of A2 and B2 resulted in highly efficient *in situ* ionization, forming counterion-free ethyl imidazolium/sulfonate ion pairs with near-quantitative conversion (>93%). This is evidenced by the complete disappearance of the neutral imidazole proton signals (see the Supporting Information for detailed peak assignment and analysis, Figures S8 and S9) and the significant shifts of the methylene protons adjacent to the end groups upon ionization. For example, the methylene protons next to the imidazole shift downfield after conversion to imidazolium (from δ_H_ 4.11 to 4.57 ppm), while those next to the sulfonate ester shift upfield (from δ_H_ 3.22 to 2.93 ppm) upon forming the sulfonate anion.^21,24,26,27^ Additionally, the integrations of the charged end-group methylene protons (peaks c′ and f′) demonstrate an approximately stoichiometric ratio of A2 to B2 in the final IAP. Furthermore, the total integration of the imidazolium ring protons (peaks d′ and d″) is approximately 3 (Figure 1d), implying the intended stoichiometry. This near-quantitative *in situ* ionization is also confirmed by FTIR spectroscopy (Figure S10), which reveals the complete disappearance of the characteristic precursor signals. Combined, the spectroscopic analyses provide compelling evidence for the efficient formation of the IAP.

**Figure 1:**
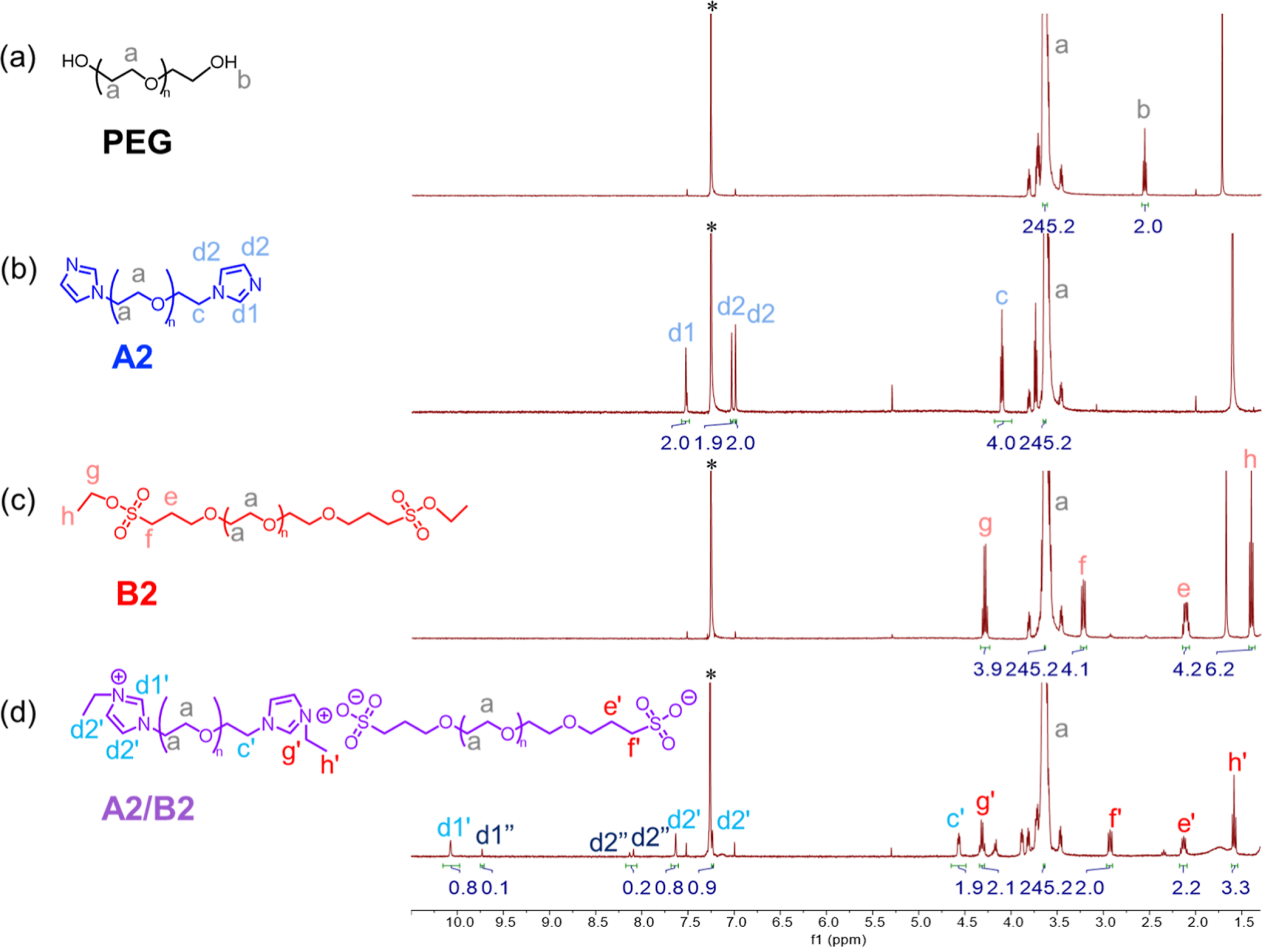
Chemical structures and ^1^H NMR spectra (in CDCl_3_*) of (a) the α,ω-diol PEG precursor, (b) A2: α,ω-imidazole PEG, (c) B2: α,ω-ethyl sulfonate PEG, and (d) the A2/B2 blend, i.e., counterion-free IAPs.

Further analysis of the ^1^H NMR spectrum of the A2/B2 blend revealed the presence of two distinct imidazolium environments, evidenced by two sets of imidazolium peaks (Figure 1d). These signals correspond to ethyl imidazolium species existing in both relatively “free” (less strongly associated) and contact ion-paired states with the sulfonate end groups. Integration of the corresponding peaks suggests a distribution of approximately 80–90% ion-paired imidazolium (d′) and ∼10–20% free imidazolium (d″). The emergence of two resolved imidazolium signals aligns with prior studies on imidazolium-based ILs, where the imidazolium proton signals are highly sensitive to local ionic interactions and hydrogen bonding.^35–37^ Many imidazolium systems exhibit fast exchange on the NMR time scale, typically resulting in only one set of averaged peaks. In contrast, the appearance of two distinct signals in our system suggests localized, restricted exchange dynamics, which can be potentially attributed to heterogeneous microenvironments. We envision that the equilibrium between close contact ion pairs and free ions is closely tied to the polarity of the medium. Accordingly, we expect the formation of contact-ion pairs to be more prevalent in the lower polarity CDCl_3_ (dielectric constant ∼4.8) solvent compared to the solvent-free polymer melt.

We corroborate our NMR findings using MALDI-TOF mass spectrometry (Figure 2), which confirms the quantitative A2 and B2 end-group functionalization and demonstrates low dispersity. Furthermore, the degree of polymerization remained invariant for both polymers compared to the precursor (DP = 62). These results are in line with the ^1^H NMR spectra (Figure S1) and SEC-MALS chromatogram (Table S5).

**Figure 2:**
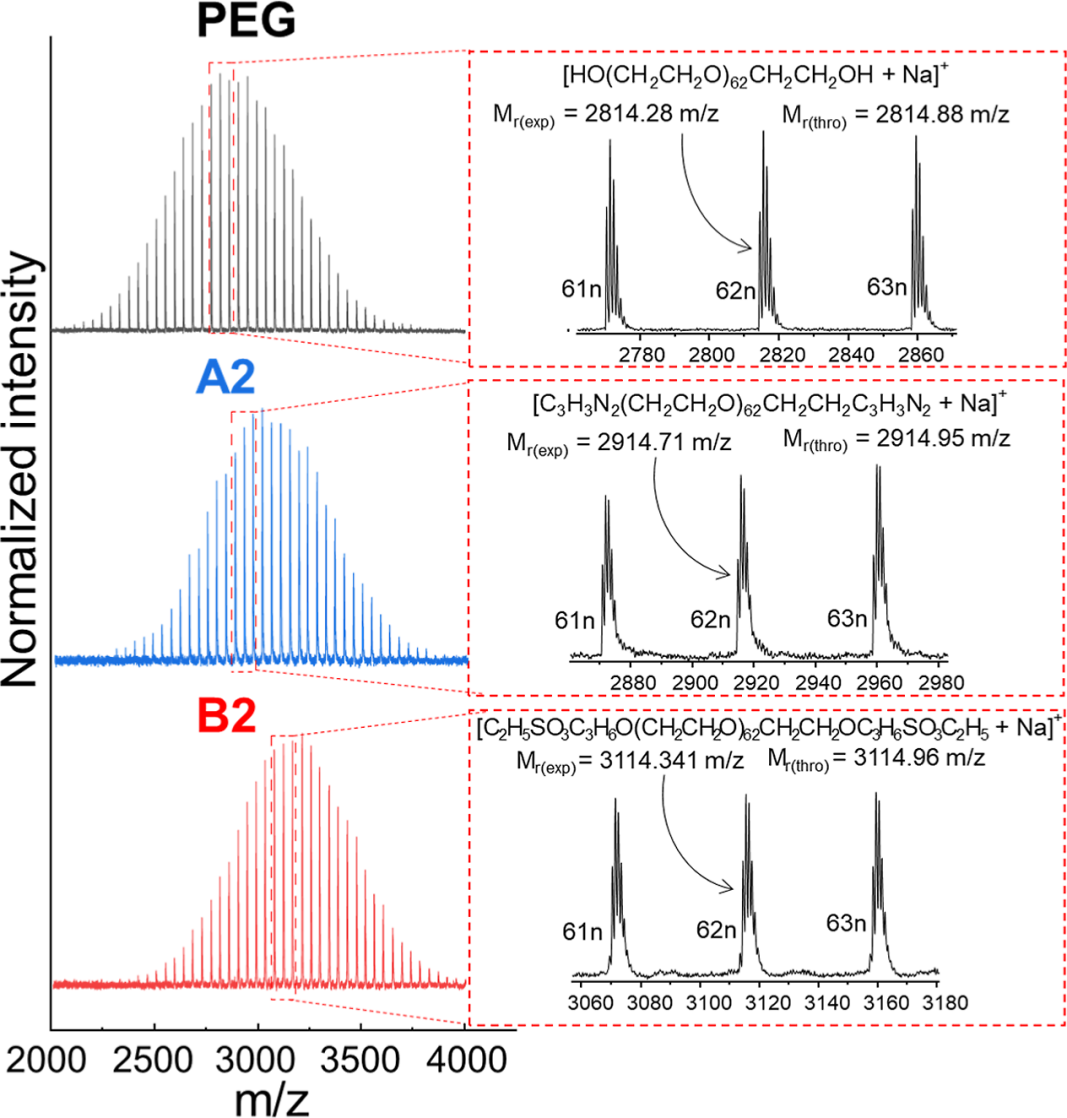
MALDI-TOF mass spectrometry (reflective mode, ionized by Na^+^) of the α,ω-diol PEG precursor, A2: α,ω-imidazole PEG, and B2: α,ω-ethyl sulfonate PEG.

### Polymer Dynamics Characterized by SAOS and PFG-NMR

We investigated the rheological properties of counterion-free IAP (A2/B2) to understand the impact of these ionic modifications. Our IAP features ionic species (both cation and anion) covalently tethered to the PEG chains. We hypothesize that the Coulombic attractions between these tethered ions lead to the formation of a transient dynamic network via chain association, thereby increasing bulk viscosity. To explore this hypothesis, we first compared the rheological performances of the unfunctionalized PEG and the A2/B2 blend. The counterion-free IAP (A2/B2 blend) demonstrates a roughly 2-fold increase in viscosity (Figure 3a), highlighting the importance of intermolecular attractions imposed by the ionic groups tethered to the chain ends. These interactions serve as reversible linkages, effectively bridging polymer chains to form an associating polymer with enhanced mechanical integrity. In the absence of small-molecule counterions that screen the polymer charges, these ionic groups engage in a stronger electrostatic association, which persists across the melt. Despite this increased viscosity, both the A2/B2 IAP and the unfunctionalized PEG behaved as Newtonian fluids above their melting temperatures. Steady shear experiments at 60 °C show that the measured viscosities are independent of the shear rate for all samples (Figure 3a) and frequency scans demonstrate the *G*″ ∝ ω^1^ power-law scaling (Figure 3b).

**Figure 3:**
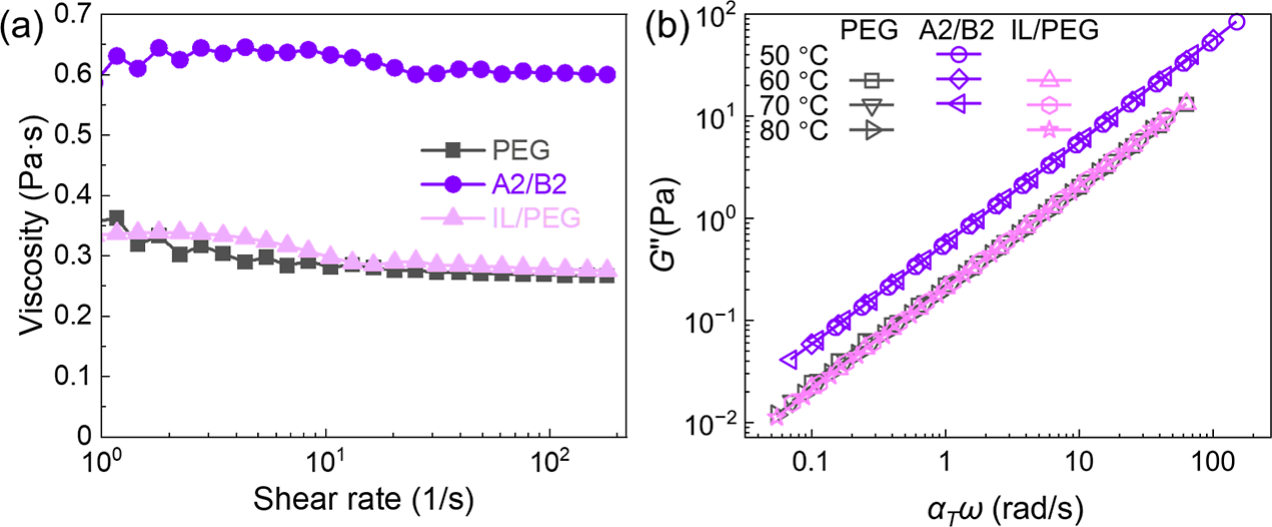
Rheology measurements for the diol PEG precursor (black symbols), A2/B2 blend (purple symbols), and IL-doped PEG (pink symbols). (a) Steady shear viscosity profiles at *T* = 60 °C, (b) frequency sweep master curves with *T*_ref_ = 60 °C. The interchain ionic interactions in A2/2B2 contribute to a higher viscosity while the mobile ion pairs in IL/PEG demonstrate a negligible effect on enhancing viscosity.

To further test the hypothesis that covalently tethered ions are crucial for enhanced viscoelastic properties, we characterized a control group (IL/PEG) prepared by blending the PEG with 1-ethyl-3-methylimidazolium methanesulfonate ([emim^+^]­[MeSO_3_^–^]). We formulated this IL/PEG blend with a molar ratio of 2:1 IL to PEG, ensuring the same overall ionic content as that in A2/B2 IAP, but featuring nontethered, mobile ions. The IL mimics the ionic junction in A2/B2 IAP. Similar to PEG and A2/B2, the IL/PEG also behaves as a Newtonian fluid above its melting temperature. However, a comparison of zero-shear viscosity reveals the order: η_0,A2/B2_ > η_0,IL/PEG_ ≈ η_0,PEG_ (Figure 3a). This order clearly demonstrates that the dynamics of the A2/B2 blend are substantially slower than those of PEG and IL/PEG. It is noteworthy that the addition of mobile IL ions to PEG leads to minimal viscosity enhancement compared to the diol PEG precursor. This indicates that the interactions between mobile IL ions and the PEG backbone are largely transient, imposing no topological constraints on the polymer chains.^38^ In other words, nontethered ions cannot interact strongly enough with PEG chains to slow down dynamics. Only when these ionic functional groups are covalently tethered to the polymer chain can an IAP form, leading to the observed increase in viscosity and mechanical integrity.

We envision that the flow in such glass-forming molecular liquids is impeded by a lack of free volume and an energy barrier to molecules sliding past one another. Thus, we fit the complex viscosity data as a function of temperature with the VFT eq (Figure 4)­

1 $$\eta \left(T\right)=\eta_{\infty }\exp\left(\frac{B}{T-T_{0}}\right)$$

where *B* is the VTF parameter, *T*_0_ is the Vogel temperature, and η_∞_ is the viscosity at infinitely high temperature. *B* and *T*_0_ of PEG have been well characterized by rheology and neutron spin echo spectroscopy (NSE).^39^ Here we fixed *T*_0_ = 155 K (appropriate for low molar mass PEG), and we treated η_∞_ and *B* as the two fitting parameters. The VFT fitting parameters summarized in Table 2 also agree well with the frequency sweep data (Figure S11), indicating that the VFT model effectively describes the viscoelastic behavior across both the temperature and frequency domains. The effective activation energy (*E*_a_) of flow in the VFT framework can be calculated by eq 2.^40^

2 $$E_{a}^{eff}\left(T\right)=R\frac{BT^{2}}{{\left(T-T_{0}\right)}^{2}}$$

**Figure 4:**
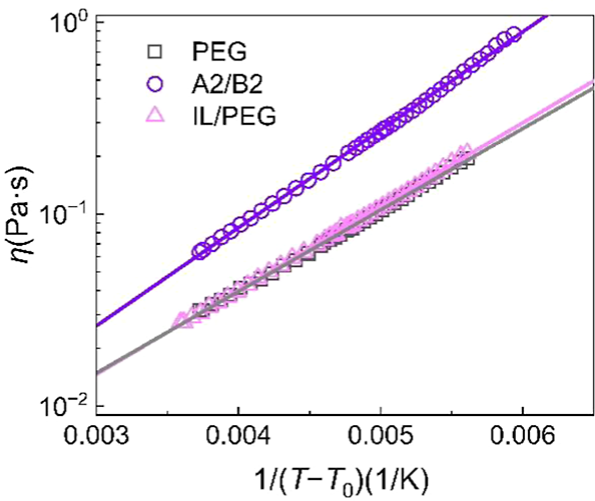
Temperature-dependent viscosity behavior of the diol PEG precursor, A2/B2 blend, and IL-doped, plotted as ln η versus 1/(*T* – *T*_0_). The solid lines represent the VFT fitting.

**Table 2:** VFT Fit of Polymer Dynamics

	η_∞_ (mPa·s)	*B* (K)	*T*_0_ (K)^a^	*E*_a_@60 °C (kJ/mol)
PEG	0.794	977	155	28.4
A2/B2 blend	0.772	1175	155	34.2
IL/PEG	0.717	1006	155	29.2

a Determined via rheology and NSE in refs 39 and 41 .

According to Table 2, the values of *E*_a_ for PEG and IL/PEG are almost identical (∼29 kJ/mol), while the A2/B2 blend activation energy is approximately 20% higher (∼34 kJ/mol). This indicates that the Coulombic interactions led to a steeper temperature dependence, rendering IAP “fragile”. Such “fragility” implies that dynamics are cooperative, requiring polymeric ions to rearrange together for motion to occur. The observed increase in *E*_a_ supports the existence of reversible, yet persistent, interchain ionic associations that constrain chain mobility across a wide temperature range. In such systems, the ionic associations act as reversible “stickers” or temporary “cross-links” with a finite lifetime, and the additional energy required for flow corresponds to the thermal energy needed to break these temporary associations.

Note that the slower dynamics in A2/B2 are not attributable to any static microstructure formation. Unlike metal–ligand or hydrogen bonding interactions in some supramolecular polymer systems, where the interaction groups microphase separate from the polymer backbone,^42–44^ the imidazolium and sulfonate groups are highly compatible with the PEG matrix. SAXS reveals a lamellar structure of PEG crystallites at room temperature, yet no nanostructure is observed in the melt states. Furthermore, WAXS indicates that the chain ends exert no influence on the crystalline structure of PEG (Figure S12). Thus, we assume all polymer samples exhibit a Gaussian-like chain configuration, whose dynamics can be captured by the Rouse framework. While our polymers (*M*_n_ ≈ 2.8–3.1 g/mol) are slightly above PEG’s entanglement molecular weight (*M*_e_ = 1.6–1.7 kg/mol^45,46^ ), they remain below the critical molecular weight for entanglement (*M*_c_ = 5–6 kg/mol^47,48^ ). Rouse dynamics are appropriate, since significant chain entanglement, impacting macroscopic properties, occurs only above *M*_c_ for PEG. The prediction for diffusion within this framework is given by eq 3 ^45^

3 $$D=\frac{k_{B}T}{N\zeta }$$

where *k*_B_ is the Boltzmann constant, *N* is the degree of polymerization, and ξ is the monomeric friction coefficient.

Similarly, the viscosity of the PEG melts can be described as

4 $$\eta =\frac{\zeta \rho N_{A}b^{2}N}{{36m}_{0}}$$

where ρ is the density (1.1 g/cm^3^), *b* is the statistical segment length (5.8 Å) of PEG,^39,49,50^ *N*_A_ is Avogadro’s constant, and *m*_0_ is the molar mass of a repeat unit. Thus, translational and relaxational dynamics should be coupled, and the products of *D* and η should be invariant with respect to chain length, as shown in eq 5.

5 $$D\eta =\frac{k_{B}T\rho N_{A}b^{2}}{{36m}_{0}}$$

The self-diffusion coefficients (*D*) and complex viscosities (η) of PEG and A2/B2 were measured at 90 °C, 75 °C, and 60 °C (Tables S3 and S4, and Figure 5a). This temperature range was selected based on the polymer’s melting point (at the low end) and by instrumental limitations of the PFG-NMR probe (at the high end). As expected, the diffusion of A2/B2 is slower than that of PEG due to interchain attractions. The calculated *D*η product for PEG is nearly temperature-independent, with a value of (6.4 ± 0.2) × 10^–13^ N. This result is in agreement with previously reported experimental data and the prediction of Rouse model (Figure 5b).^51^

**Figure 5:**
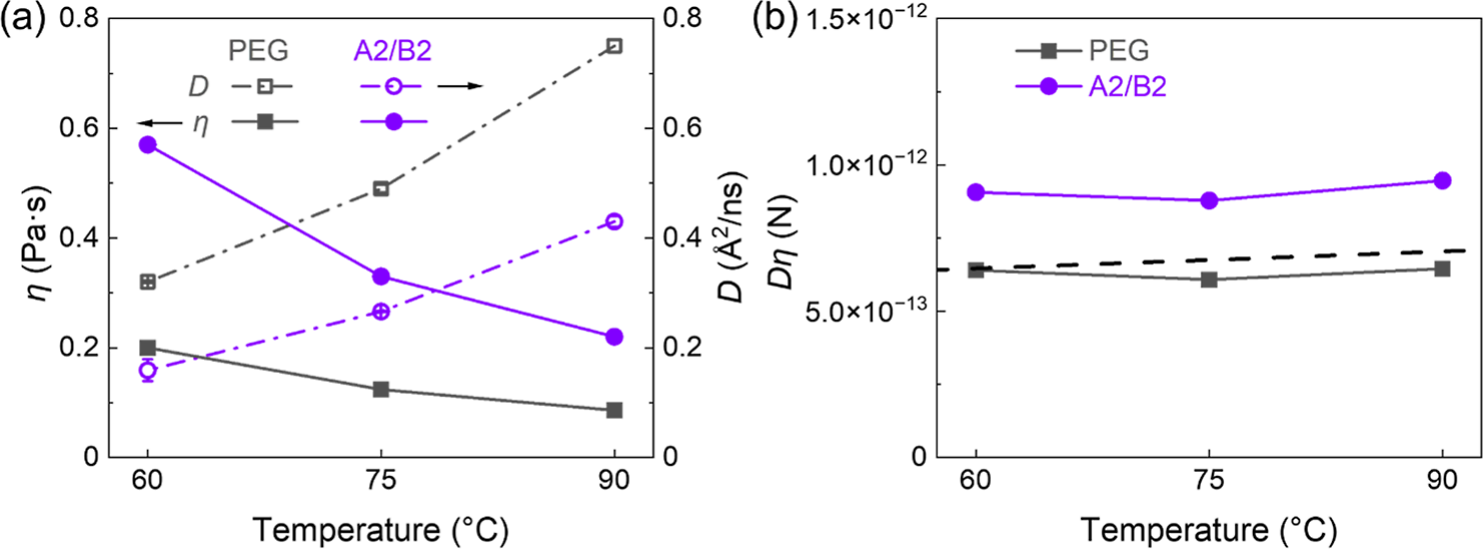
(a) Complex viscosity (solid symbols) and self-diffusion coefficient (open symbols) profiles of PEG (block squares) and A2/B2 (purple circles), with error bars shown for the diffusion data. (b) Products of *D* and η of PEG (black squares) and A2/B2 blend (purple circles). The dashed line represents the Rouse prediction (eq 5).

In contrast, the *D*η product for the A2/B2 blend, while also weakly temperature dependent, deviates from the Rouse prediction by almost 50% across the entire experimental temperature range. We speculate that this deviation in the IAP system arises from the distinct ways transient ionic associations influence macroscopic viscous flow and microscopic chain diffusion. Macroscopic stress relaxation, which governs viscosity (η), requires the dissociation of ionic bonds and subsequent recombination with new chains to allow for full topological rearrangement.^52^ As a result, the reversible ionic associations act as temporary cross-links that impede bulk flow. In the meantime, microscopic chain diffusion (*D*) reflects the center-of-mass motion of individual chains. While ionic associations do hinder this motion, individual chains can still undergo relatively rapid local “hopping”. This involves transient disengagement from current partners and exploration of the immediate surroundings before potentially reforming either the original bond or forming a new one.^52–55^ Such local escape and motion of individual chains occur on a faster time scale than the global network relaxation. The latter requires the breaking of original associations and the formation of new ones to relieve macroscopic stress.^52^ This differential impact on global network relaxation (viscosity) versus localized individual chain mobility (diffusion) leads to an elevated *D*η product compared to the Rouse prediction. A similar behavior has been observed in other associating polymers due to this chain mobility/viscosity decoupling.^53–55^ Future work focusing on quantifying the time scales of ion hopping events in IAPs and the association–dissociation equilibrium, using broadband dielectric spectroscopy,^56,57^ would be highly valuable, as it would enable a more detailed molecular understanding of the interplay between microscopic chain mobility and macroscopic viscoelastic behavior.

## Conclusions and Outlook

In this work, we have demonstrated an innovative and straightforward method for synthesizing IAPs (poly^+^/poly^–^) without introducing superfluous counterions via *in situ* ionization and coupling of sulfonate-alkyl precursors. A key innovation in preparing these IAP systems is our development of a base-free salt metathesis route for the synthesis of telechelic PEG-ethyl sulfonate (B2). This route achieved nearly quantitative chain-end fidelity (>97%), overcoming the low fidelity (<80%) typically obtained by conventional methods. Reactive blending of telechelic PEG-imidazole (A2) and the high-chain-end-fidelity PEG-sulfonate ester (B2) in the melt state achieved quantitative ionization (>93%). The ionic junctions between PEG building blocks increase the interchain friction, leading to 100% increase in viscosity compared to the PEG building block, which is indicative of strong interchain associations. Furthermore, the A2/B2 blend dynamics deviate from Rouse predictions, consistent with the formation of a transient dynamic network and decoupled chain mobility.

This counterion-free synthetic approach offers significant advantages for advancing fundamental polymer science and opens new avenues for materials design. The lack of screening ions in our system ensures strong, unscreened interchain Coulombic interactions, which are crucial for controlling and observing electrostatically stabilized microphases.^58–60^ Research indicates that the introduction of mobile counterions can suppress microphase formation and induce macroscopic phase separation.^58,61,62^ Our counterion-free system provides an ideal platform to investigate the intrinsic physics of electrostatic self-assembly. This allows for a precise understanding of interaction strength and microphase evolution, potentially leading to smaller microphase domain sizes, more ordered local structures, and sharper interfaces.

In IAPs, the Coulombic interaction is given by $E=k_{B}T\frac{l_{B}}{r}$ , where *r* is the distance between opposite charges and *l*_B_ is the Bjerrum length, which is inversely proportional to the dielectric constant (ε) of the polymer matrix. We further hypothesize that these interchain interactions would be significantly enhanced in less polar polymer matrixes compared to the relatively polar (ε ≈ 7.5) PEG matrix. Looking forward, we envision that the modular nature of our synthesis route and *in situ* ionization strategy can be readily adapted to other polymers, allowing for straightforward tuning of ionic association strength and thus the mechanical properties. By simply selecting different neutral nucleophiles (e.g., amine and pyridine) or incorporating these groups onto various polymer backbones (e.g., poly cyclooctene and polystyrene) or architectures (e.g., multiarm and block copolymers), a vast range of ion-associated polymers (IAPs) can be accessed. This modular approach will open a vast design space of IAPs that features enhanced tunability and the ability to be assembled/disassembled on demand, ultimately offering alternatives to conventional plastics with improved recyclability and enhanced mechanical properties.

## Supplementary Material



## Notes

Synthetic procedures, NMR spectra, MALDI-TOF spectra, SAOS, SAXS/WAXS, PFG-NMR, DSC, TGA, and SEC-MALS analyses (PDF)
