# Supplementary material for: Counterion-Free Ionic Associating Polymers: In Situ Ionization and Coupling of Alkyl Sulfonate Precursors
Source: Macromolecules. 2025 Aug 25;58(20):11315–23. doi: 10.1021/acs.macromol.5c01487 (PMC12573802; doi:10.1021/acs.macromol.5c01487)
Supplement: Supplementary file 1 [file ma5c01487_si_001.pdf]

Supporting information for

Counterion-Free Ionic Associating Polymers: *In-Situ* Ionization and Coupling of Alkyl Sulfonate Precursors

Jie Xu,[1] Chia-Chi Tsai,[1] Oscar Nordness,[*2] Shuyi Xie[*1]

[1]Department of Chemical Engineering, Texas A&M University, College Station, TX 77843, United States

[2]Henry Krumb School of Mines, Earth and Environmental Engineering Department, Columbia University, New York, NY10027, United States

E-mail: shuyixie@tamu.edu

Table of Contents

Section A. Sample Preparation **S3**

(1) Preparation of α,ω-imidazole PEG (A2) S3

(2) Preparation of α,ω- ethyl sulfonate PEG (B2) S4

(3) Preparation of counterion-free A2/B2 blend S7

(4) Preparation of IL doped PEG diol (IL/PEG) S8

Section B. Characterization Details and Data **S9**

(1) Proton nuclear magnetic resonance (^{1}H NMR) spectroscopy S9

(2) Fourier transform infrared (FTIR) spectroscopy S20

(3) Matrix-assisted laser desorption/ionization time of flight (MALDI-TOF) mass spectroscopy S21

(4) Small-amplitude oscillatory shear (SAOS) tests S22

(5) Small- and wide-angle X-ray scattering (SAXS/WAXS) S24

(6) Pulsed field gradient (PFG-NMR) spectroscopy S26

(7) Differential scanning calorimetry (DSC) S29

(8) Thermogravimetric analysis (TGA) S32

(9) Size exclusion chromatography equipped with multi-angle light scattering (SEC-MALS) S33

Section C. References **S35**

Section A. Sample Preparation

(1) Preparation of α,ω-imidazole PEG (A2)

- **Synthesis of α,ω-mesylate PEG (A2 precursor)**

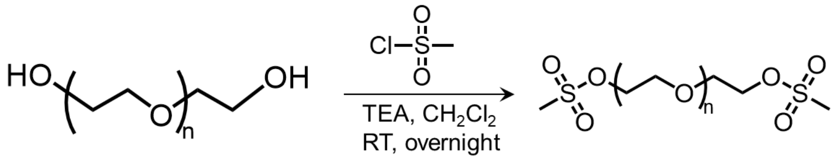

Synthesis of α,ω-imidazole PEG (A2) was carried out starting from α,ω-diol PEG precursor. 3 g of purified diol PEG (1 mmol) was dissolved in 15 mL of anhydrous DCM in a 100 mL round bottom flask. 0.8 g of triethylamine (8 mmol) was added into diol PEG/DCM solution while stirring. The solution was purged with nitrogen for 30 minutes and then cooled to 0 °C in an ice bath. Subsequently, 0.92 g of MsCl (8 mmol) was add dropwise to the mixture, and the ice bath was removed. The resulting mixture was stirred at room temperature overnight. Following the reaction, 30 mL of H_2O was added to the polymer solution. The mixture was vortexed for 1 minute and centrifuged at 3500 rpm for 5 minutes. The aqueous phase (top) was discarded, and the organic phase (bottom) was collected. The transparent organic phase was precipitated in cold diethyl ether (1:5 volume ratio). The collected solid was dried in a vacuum oven (50 mtorr) at room temperature for 48 hours. This yielded white powder α,ω-mesylate PEG (2.31 g, 77% yield) with nearly quantitative chain-end fidelity (> 98%).

- **Synthesis of α,ω-imidazole PEG (A2)**

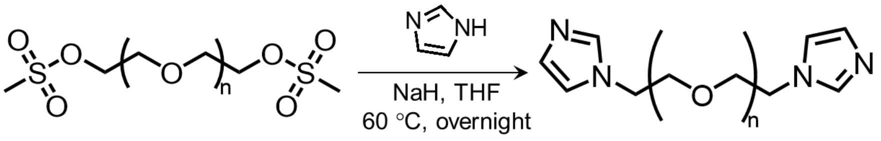

2 g of α,ω-mesylate PEG (0.67 mmol, from last step) was dissolved in 8 mL of anhydrous THF in a 25 mL round bottom flask. The solution was purged with nitrogen for 30 minutes. In a glovebox, 0.064 g of NaH (2.67 mmol) and 0.18 g of imidazole (2.67 mmol) was added in a 100

mL Schlenk flask. After the Schlenk flask containing NaH and imidazole was connected to the Schlenk line, 4 mL of anhydrous THF was added to the Schlenk flask. It accompanied by the evolution of hydrogen gas, which was released through the Schlenk line. After that, the α,ω-mesylate PEG/THF solution from the 25 mL round bottom flask was transferred dropwise into the stirred imidazole/NaH/THF suspension in the 100 mL Schlenk flask. The resulting mixture was stirred at 60 °C overnight in an aluminum bead bath (Lab Armor). After cooling to room temperature, the reaction was terminated by the dropwise addition of 1 mL of MeOH. The polymer product was purified by precipitation in a cold diethyl ether (1:5 volume ratio) twice. The collected solid was dried in a vacuum oven (50 mtorr) at room temperature for 24 hours, followed by drying at 60 °C for an additional 24 hours. This yielded white powder α,ω-imidazole PEG (A2) (1.74 g, 75% yield) with nearly quantitative chain-end fidelity (> 98%).

(2) Preparation of α,ω-ethyl sulfonate PEG (B2)

- **Synthesis of α,ω-sodium sulfonate PEG (B2 precursor)**

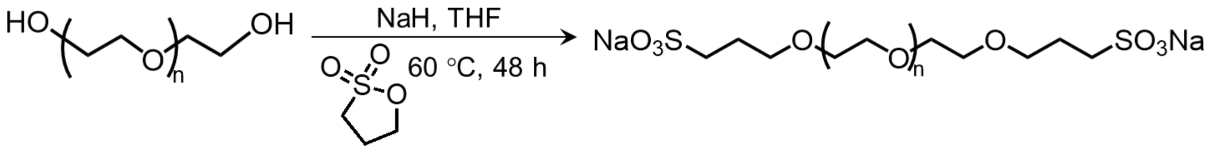

To prepare B2 precursor (α,ω-sodium sulfonate PEG), we started from α,ω-diol PEG precursor. 12 g of purified α,ω-diol PEG (4 mmol) was dissolved in 40 mL of anhydrous THF in a 100 mL round bottom flask and purged with nitrogen for 30 minutes. In a glovebox, 0.288 g of NaH (12 mmol) was added to a 250 mL round bottom flask. After connecting the 250 mL round bottom flask to the Schlenk line, 5 mL of anhydrous THF was added to the Schlenk flask containing NaH, accompanied by the evolution of hydrogen gas, which was released through the Schlenk line. Subsequently, the α,ω-diol PEG/THF solution from the 100 mL round bottom flask was transferred dropwise into the stirred NaH suspension in the 250 mL round bottom flask. The resulting mixture was stirred at room temperature for 15 minutes. Following this, a solution of 1.5 g of propane sultone (12 mmol) in 5 mL of anhydrous THF was added into the 250 mL round bottom flask. The reaction mixture was stirred at 60 °C for 48 hours in an aluminum bead bath

(Lab Armor). Following the reaction, the mixture was cooled down to room temperature. Anhydrous isopropanol (1 mL) was added, and the mixture was stirred for 10 minutes. The polymer solution was precipitated in a cold diethyl ether (1:5 volume ratio). The precipitated product was collected by filtration. The collected solid was washed with isopropanol. The purified α,ω-sodium sulfonate PEG (B2 precursor) was dried in a vacuum oven (50 mtorr) at room temperature for 24 hours, followed by drying at 60 °C for an additional 24 hours. This yielded α,ω-sodium sulfonate PEG (B2 precursor) as a white powder (11.83 g, 99% yield) with nearly quantitative chain-end fidelity (> 99%).

- **Synthesis of α,ω-sulfonate ester PEG (B2) in basic condition (conventional route)**

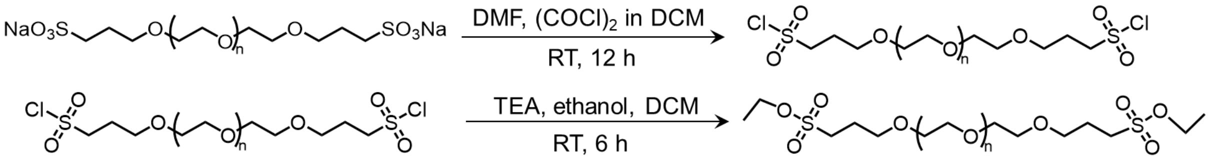

The conventional route to get α,ω-sulfonate ester PEG (B2) went thought α,ω-sulfonyl chloride PEG as intermediate and using ethanol/triethylamine solution for esterisfication. 1 g of α,ω-sodium sulfonate PEG (B2 precursor, 0.33 mmol) was added to a 25 mL Schlenk flask and dried using Schlenk line under vacuum (50 mtorr) at the 80 °C for overnight to remove residual traces of water. All subsequent manipulations before precipitation were performed in a glovebox under an inert atmosphere. 5 mL anhydrous DMF was added into the 25 mL Schlenk flask containing the dried PEG sulfonate. The resulting solution was then pre-cooled in a freezer inside the glovebox. In a separate 25 mL round bottom flask, 0.42 g of $(COCl)_2$ (3.3 mmol) was dissolved in 1 mL of anhydrous DCM and also pre-cooled in the glovebox freezer. The $(COCl)_2$/DCM solution was slowly transferred into the stirred Schlenk flask containing the PEG sulfonate solution. Instantaneous gas release (CO_2/CO) was observed upon formation of the Vilsmeier reagent. The reaction mixture was stirred at room temperature for 12 hours. Following the reaction, the product was precipitated in cold diethyl ether (1:5 volume ratio). The precipitated product was collected

by filtration. The collected solid (α,ω- sulfonyl chloride PEG) was transferred to a 25 mL Schlenk flask and dried under vacuum (50 mtorr) *via* the Schlenk line at room temperature for 1 hour.

All steps in this synthesis (addition of DCM, cooling, solution preparation, transfer, stirring, and NMR monitoring) were performed in a glovebox under an inert atmosphere. Following the drying of the intermediate, 5 mL of anhydrous DCM was added to a 100 mL Schlenk flask containing the dried α,ω-sulfonyl chloride PEG (~1 g, 0.33 mmol) from the previous step. The resulting mixture was then pre-cooled in a freezer inside a glovebox. In a separate 25 mL round bottom flask, a solution of 0.152 g ethanol (3.3 mmol) and 0.134 g triethylamine (1.32 mmol) was prepared in 1 mL of anhydrous DCM and also pre-cooled in the glovebox freezer. On a glovebox, the pre-cooled sulfonyl chloride PEG/DCM mixture was slowly transferred into the stirred solution of ethanol and TEA in the 25 mL round bottom flask. The resulting reaction mixture was stirred at room temperature for 6 hours in a glovebox. Conversion was monitored by ^{1}H NMR spectroscopy. After the reaction, volatile components (solvent, remaining ethanol and TEA) were removed under vacuum (50 mtorr) using a Schlenk line at room temperature for 24 hours. This yielded α,ω-sulfonate ester PEG (B2) as a light-yellow powder (0.2 g, 20% yield) with functionality less than 80%.

- **Synthesis of α,ω-sulfonate ester PEG (B2) in neutral condition (this work)**

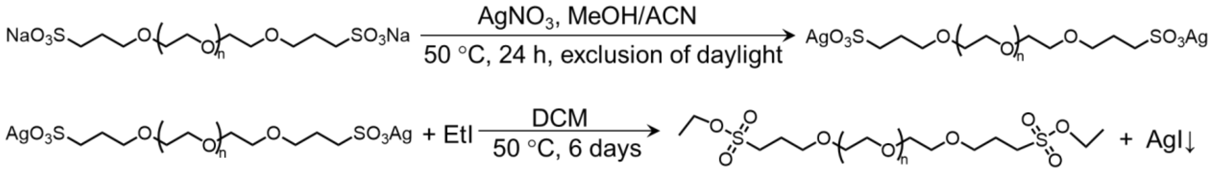

The new synthesis route to get α,ω-sulfonate ester PEG (B2) in this work employed a salt metathesis nucleophilic substitution reaction in neutral reaction condition. 1 g of α,ω-sodium sulfonate PEG (B2 precursor, 0.33 mmol) was added to a 25 mL Schlenk flask and dried using Schlenk line under vacuum (50 mtorr) at the 80 °C for overnight to remove residual traces of water. 2 mL of MeOH was added to the 25 mL Schlenk flask containing the dried PEG sulfonate. In a separate 100 mL Schlenk flask, 1.14 g of $AgNO_3$ (6.67 mmol) was dissolved in 5 mL of ACN.

Both solutions were subjected to three freeze-pump-thaw cycles. In a glovebox, the $AgNO_3$/ACN solution was heated to 60 °C in an aluminum bead bath (Lab Armor). The PEG-SO_3Na/MeOH solution was added dropwise to the heated $AgNO_3$/ACN solution. The mixture was reacted for 24 hours under strict exclusion of daylight in a glovebox. After reaction, solvent was removed under vacuum using a Schlenk line at room temperature for 48 hours. This yielded a light-yellow powder containing α,ω-sulfonate silver PEG potentially mixed with $AgNO_3$.

In a glovebox under an inert atmosphere, 25 mL of anhydrous DCM was added to a 100 mL Schlenk flask containing dried α,ω-sulfonate silver PEG (~1 g, 0.33 mmol) and potential $AgNO_3$ from the previous step. After dissolution, 1 mL of EtI (12.43 mmol) was added dropwise into the stirred PEG-SO_3Ag solution. The reaction was carried out for 6 days at 50 °C with a condenser in the glovebox. After reaction, the light-yellow suspension was centrifuged to remove the precipitates. The transparent polymer solution was then extracted with diethyl ether (1:10 volume ratio). The resulting polymer solution in diethyl ether was collected and dried by rotavapor outside the glovebox. The resulting solid was further dried under vacuum (50 mtorr) using Schlenk line at room temperature for 48 hours to remove residual volatile components. The light-yellow powder obtained was redissolved in anhydrous EtOH (2 wt%). The resulting solution was then recrystallized at −25°C in freezer inside the glovebox. The precipitated white solid was collected and dried under vacuum using a Schlenk line at room temperature for 48 hours. This yielded α,ω-sulfonate ester PEG (B2) as a white powder (0.25 g, 25% yield) with nearly quantitative chain-end fidelity (> 97%). The low yield is attributed to significant polymer loss (exceeding 50%) during the diethyl ether extraction and anhydrous ethanol recrystallization steps.

(3) Preparation of counterion-free A2/B2 blend

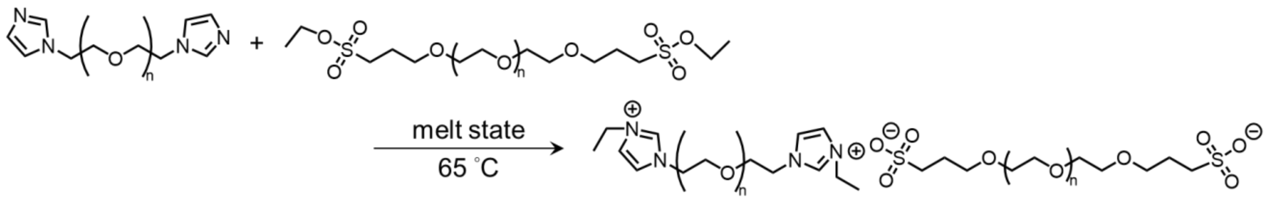

α,ω-imidazole PEG (A2) and α,ω-sulfonate ester PEG (B2) was separately dried under vacuum (50 mtorr) at 50 °C for 30 minutes using a Schlenk line. After that, equal molar amounts of A2 (0.11 g, 0.04 mmol) and B2 (0.11 g, 0.04 mmol) were weighed and placed in a 25 mL round bottom flask. In a glovebox, the mixture of A2 and B2 was heated to 65 °C and stirred for 48 hours in an aluminum bead bath (Lab Armor), without using any solvent. This *in-situ* ionization yielded the counter-ion free ionic associating polymer, forming ethyl imidazolium/sulfonate ion pairs with near-quantitative conversion (> 93%).

(4) Preparation of IL doped PEG diol (IL/PEG)

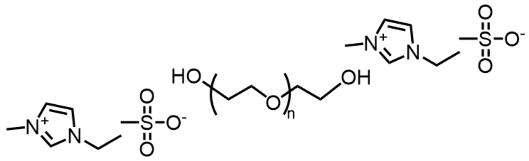

1 g of purified α,ω-diol PEG (0.36 mmol) and 0.15 g of [$emim^+$][$MeSO_3^-$] (IL) were weighted and placed in a 25 mL round bottom flask. In a glovebox, the mixture of diol PEG and IL was heated to 65 °C and stirred for 48 hours in an aluminum bead bath (Lab Armor).

Section B. Characterization Details and Data

(1) Proton nuclear magnetic resonance (^{1}H NMR) spectroscopy

^{1}H NMR spectroscopy was obtained from a Bruker Avance Neo 400Hz spectrometer. Chloroform-d (≥ 99.8 atom %D, anhydrous) was selected as the NMR solvent to ensure optimal polymer solubility and to prevent peak overlay. All NMR tests were conducted for 16 scans with a delay relaxation time of 15 seconds between each scan at 25 °C.

- **^{1}H NMR data for compounds in the synthesis of A2**

Figure S1 shows the ^{1}H NMR spectrum of α,ω-diol PEG. The resonance at 2.56 ppm (peak b) is assigned as the protons of the terminal hydroxyl groups. The prominent resonance at 3.64 ppm (peak a) corresponds to the methylene protons of the repeating ethylene glycol units in the PEG backbone. By setting the integral of b to 2 (representing the two terminal protons), the integral of peak a is 245.2, which yields a degree of polymerization (n) of 61.3 (245.2/4 protons per repeat unit). Due to its distinct and well-defined nature, the resonance of the repeating ethylene glycol units (peak a) was used as an internal reference for calculating chain-end fidelity and conversion in all functionalized PEG derivatives and the IAP blend. The validity of this method was confirmed by ^{1}H NMR experiment using an internal standard, which verified the integral of peak a is unchanged upon functionalization and blending.

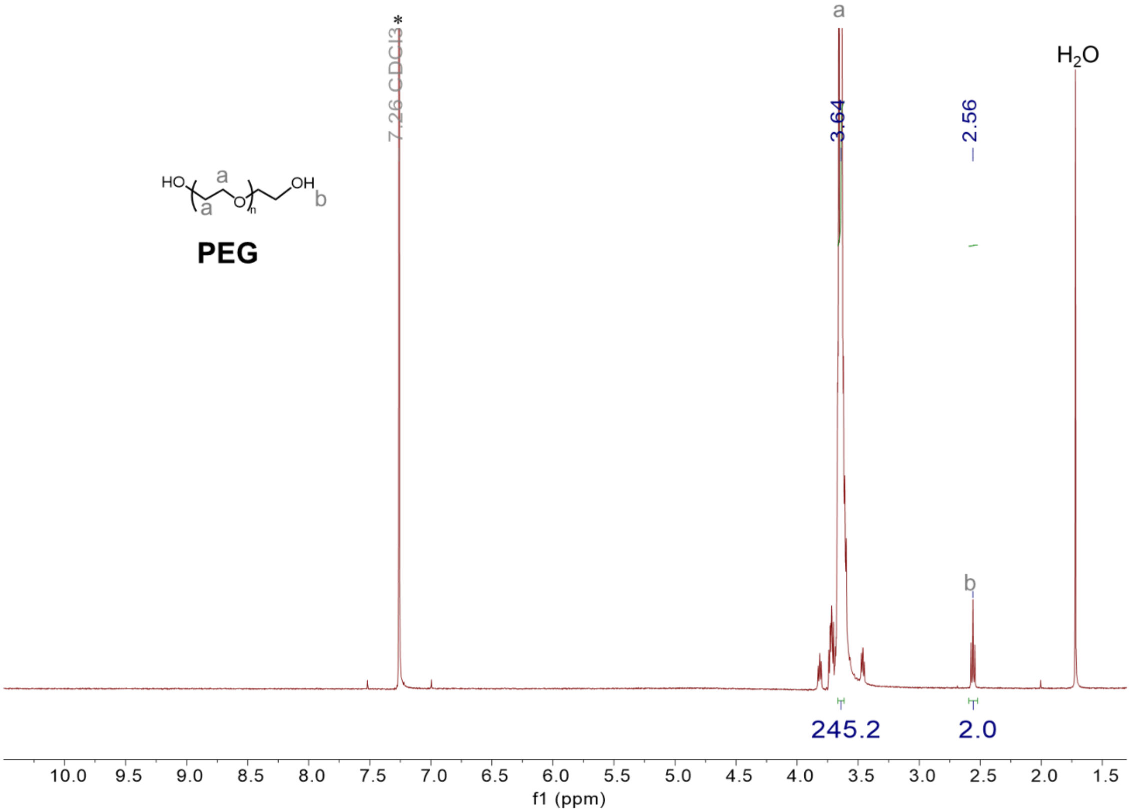

Figure S1. ^{1}H NMR spectrum (in $CDCl_3^*$) of α,ω-diol PEG.

Figure S2 shows ^{1}H NMR spectroscopy of α,ω-mesylate PEG after purification and drying. The resonance at 4.38 ppm (peak i) is assigned as the methylene protons adjacent to the mesylate end-groups. The resonance at 3.08 ppm (peak j) is assigned to the terminal methyl groups. Using the integral of the repeating ethylene glycol units (peak a) as a reference (245.2), the integrals of peaks i and j were found to be 4.0 and 5.9, respectively. The near nearly quantitative chain-end fidelity of the α,ω-mesylate PEG was confirmed by the complete disappearance of peak b (corresponding to the terminal hydroxyl groups of diol PEG precursor) and the observed integrals of peak i (4.0, expect 4.0) and j (5.9, expect 6.0).

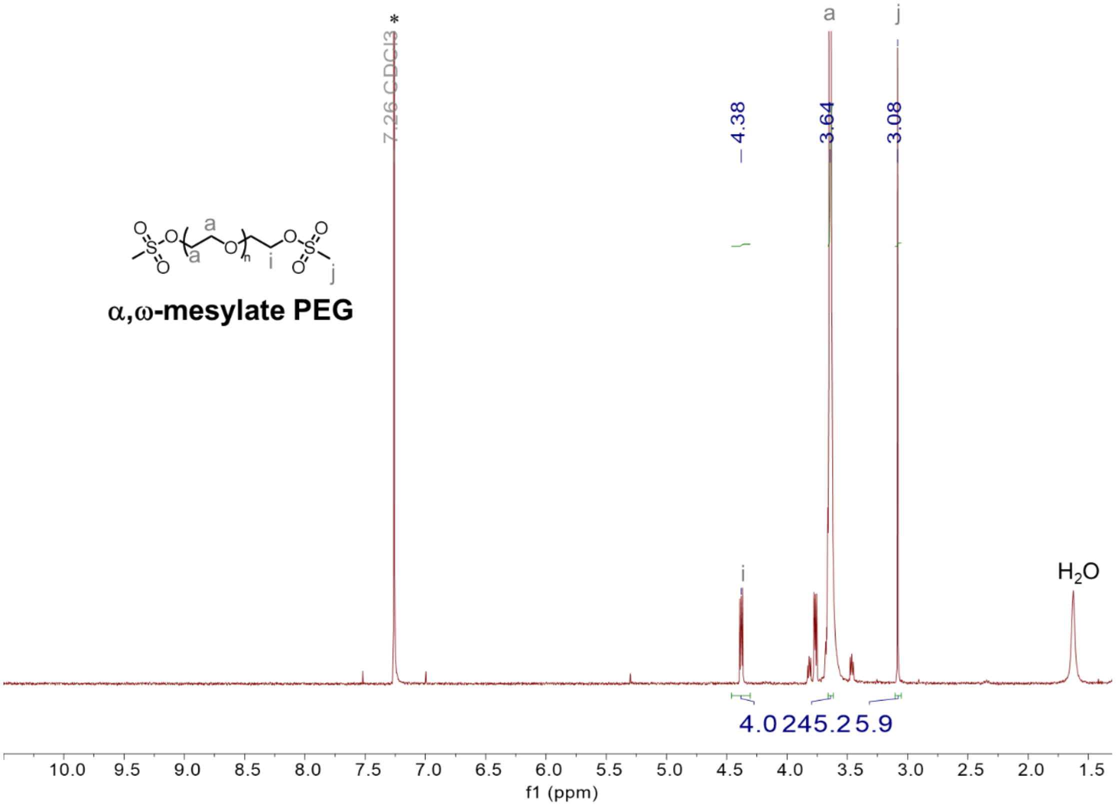

Figure S2. *^{1}H NMR spectrum (in $CDCl_3$*) of α,ω-mesylate PEG.*

Figure S3 shows ^{1}H NMR spectroscopy of α,ω-imidazole PEG (A2) after purification and drying. The resonance at 4.11 ppm (peak c) is assigned as the methylene protons adjacent to the imidazole end-groups. The resonances at 7.53 ppm, 7.04 ppm and 6.99 ppm (peak d1 and d2) are assigned to the terminal imidazole groups. Using the integral of the repeating ethylene glycol units (peak a) as a reference (245.2), the integrals of peaks c, d1, and two d2 peaks were found to be 4.0, 2.0, 1.9 and 2.0, respectively. The near nearly quantitative chain-end fidelity of the α,ω-imidazole PEG was confirmed by the complete disappearance of peaks i and j (corresponding to the terminal mesylate groups of α,ω-mesylate PEG precursor) and the observed integrals of peak c (4.0, expect 4.0), d1 (2.0, expected 2.0) and d2 (1.9 + 2.0 = 3.9, expected 4.0).

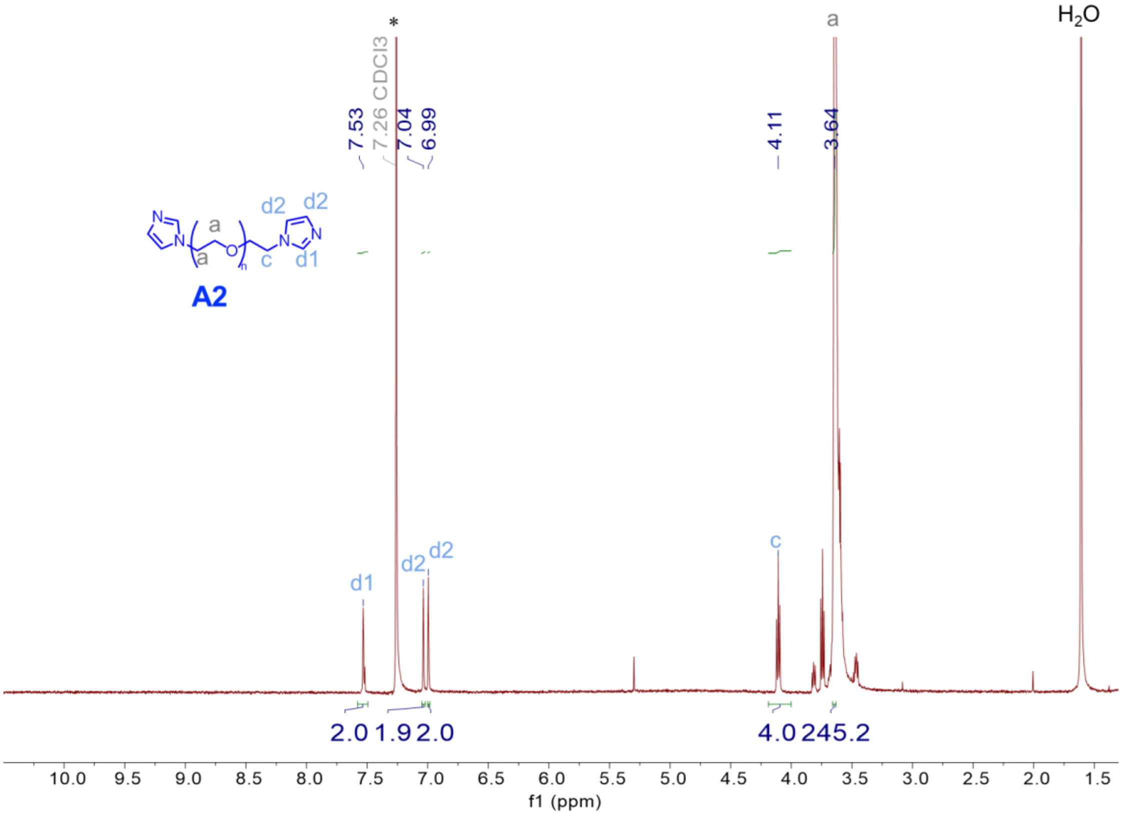

Figure S3. ^{1}H NMR spectrum (in $CDCl_3$*) of α,ω-imidazole PEG(A2).

- **^{1}H NMR data for compounds in the synthesis of α,ω-sulfonate ester PEG (B2)**

Figure S4 shows ^{1}H NMR spectroscopy of α,ω-sodium sulfonate PEG after purification and drying. The resonances at 2.87 ppm (peak l) and 2.04 ppm (peak k) are assigned as the methylene protons directly adjacent and beta to the sodium sulfonate end-groups, respectively. Using the integral of the repeating ethylene glycol units (peak a) as a reference (245.2), the integrals of peaks l and k were found to be 4.0 and 4.0, respectively. The near nearly quantitative chain-end fidelity of the α,ω-sodium sulfonate PEG was confirmed by the complete disappearance of peak b (corresponding to the terminal hydroxyl groups of α,ω-diol PEG precursor) and the observed integrals of peak l (4.0, expect 4.0) and k (4.0, expected 4.0).

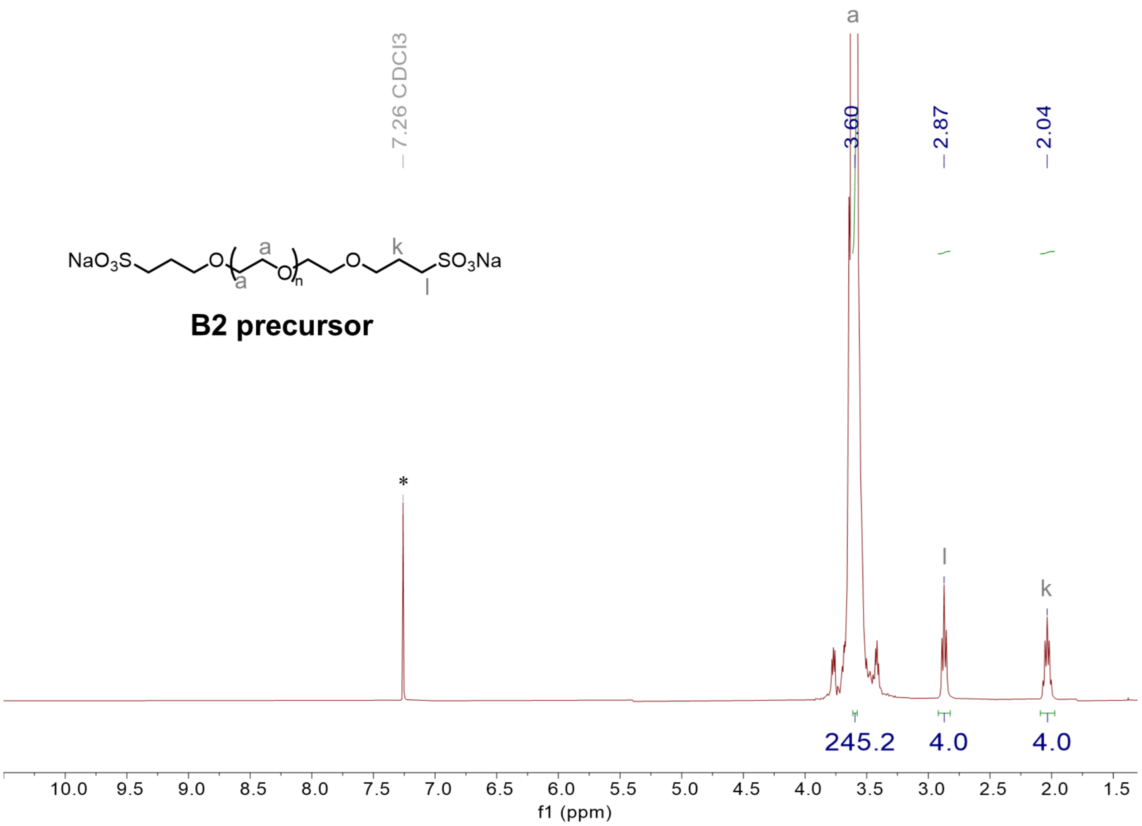

Figure S4. [1]*H NMR spectrum (in* $CDCl_3{}^{*}$*) of α,ω-sodium sulfonate (B2 precursor).*

Figure S5 shows ^{1}H NMR spectroscopy of α,ω-sulfonyl chloride PEG after purification and drying. The resonance at 2.28 ppm (peak m) is assigned as the methylene protons directly adjacent to the sulfonyl chloride end-groups, respectively. Using the integral of the repeating ethylene glycol units (peak a) as a reference (245.2), the integrals of peaks m was found to be 3.6. The chain-end fidelity of the α,ω- sulfonyl chloride PEG was estimated to be only approximately 90%, based on the incomplete disappearance of peaks k and l (corresponding to the methylene protons adjacent to terminal groups of α,ω-sodium sulfonate PEG precursor) and the observed integral of peak m (3.6, expect 4.0).

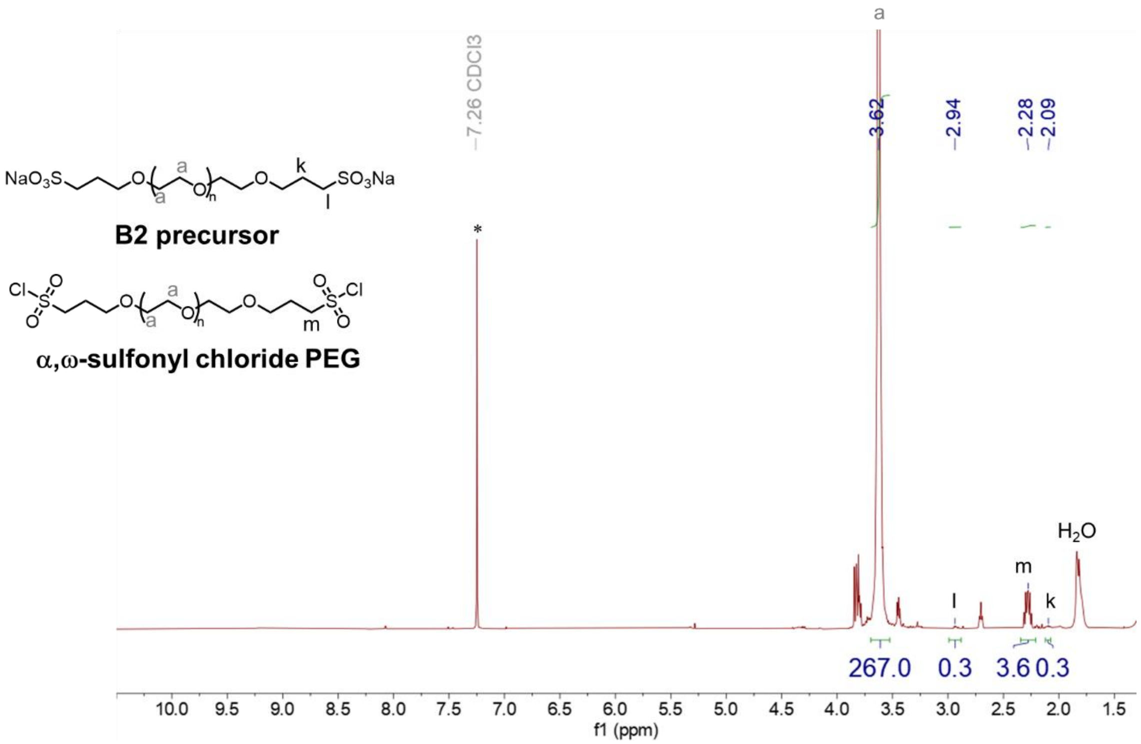

Figure S5. ^{1}H NMR spectrum (in $CDCl_3$) of α,ω- sulfonyl chloride PEG.*

Figure S6 shows ^{1}H NMR spectroscopy of α,ω-sulfonate ester PEG (B2) prepared by a conventional route under basic conditions, without further purification. The resonance at 4.28 ppm (peak g) and 1.39 ppm (peak h) are assigned to the methylene and methyl protons of ethyl groups, respectively. The resonances at 3.21 ppm (peak f) and 2.10 ppm (peak e) are assigned to the methylene protons directly adjacent and beta to the sulfonate ester end-groups, respectively. Using the integral of the repeating ethylene glycol units (peak a) as a reference (245.2), the integrals of peaks g, f e and h was found to be 3.1, 3.0, 3.9 and 5.2, respectively. The chain-end fidelity of the α,ω-sulfonyl chloride PEG was estimated to be only approximately 75%, based on the observed integral of peak g (3.1, expect 4.0) and f (3.0, expect 4.0).

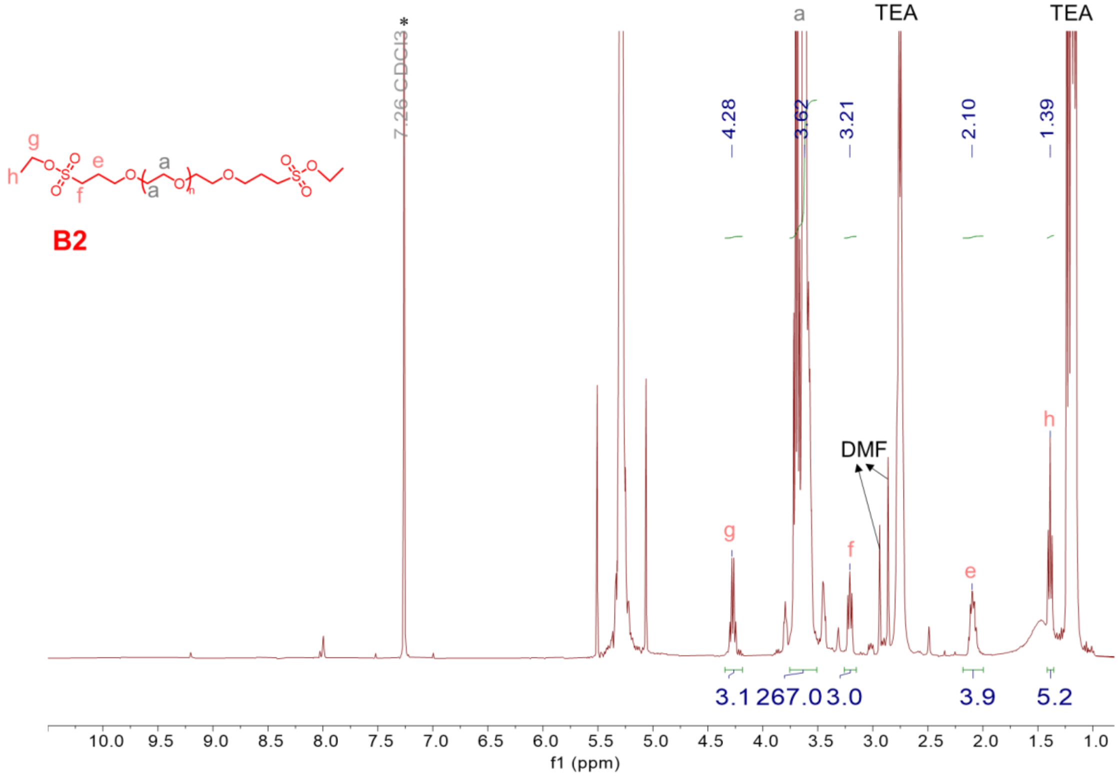

Figure S6. *¹H NMR spectrum (in $CDCl_3$*) of α,ω-sulfonate ester PEG (B2) prepared by a conventional route under basic conditions, illustrating a functionality yield below 80%.*

Figure S7 shows the ¹H NMR spectrum of α,ω-sulfonate ester PEG (B2) prepared by a new synthesis route under neutral conditions after purification and drying. The resonance at 4.30 ppm (peak g) and 1.41 ppm (peak h) are assigned to the methylene and methyl protons of ethyl groups, respectively. The resonances at 3.22 ppm (peak f) and 2.11 ppm (peak e) are assigned to the methylene protons directly adjacent and beta to the sulfonate ester end-groups, respectively. Using the integral of the repeating ethylene glycol units (peak a) as a reference (245.2), the integrals of peaks g, f, e and h were found to be 3.9, 4.1, 4.2 and 6.2, respectively. The nearly quantitative chain-end fidelity of the α,ω-sodium sulfonate PEG was confirmed by the observed integrals of peak g (3.9, expect 4.0), f (4.1, expected 4.0), e (4.2, expected 4.0) and h (6.2, expected 6.0).

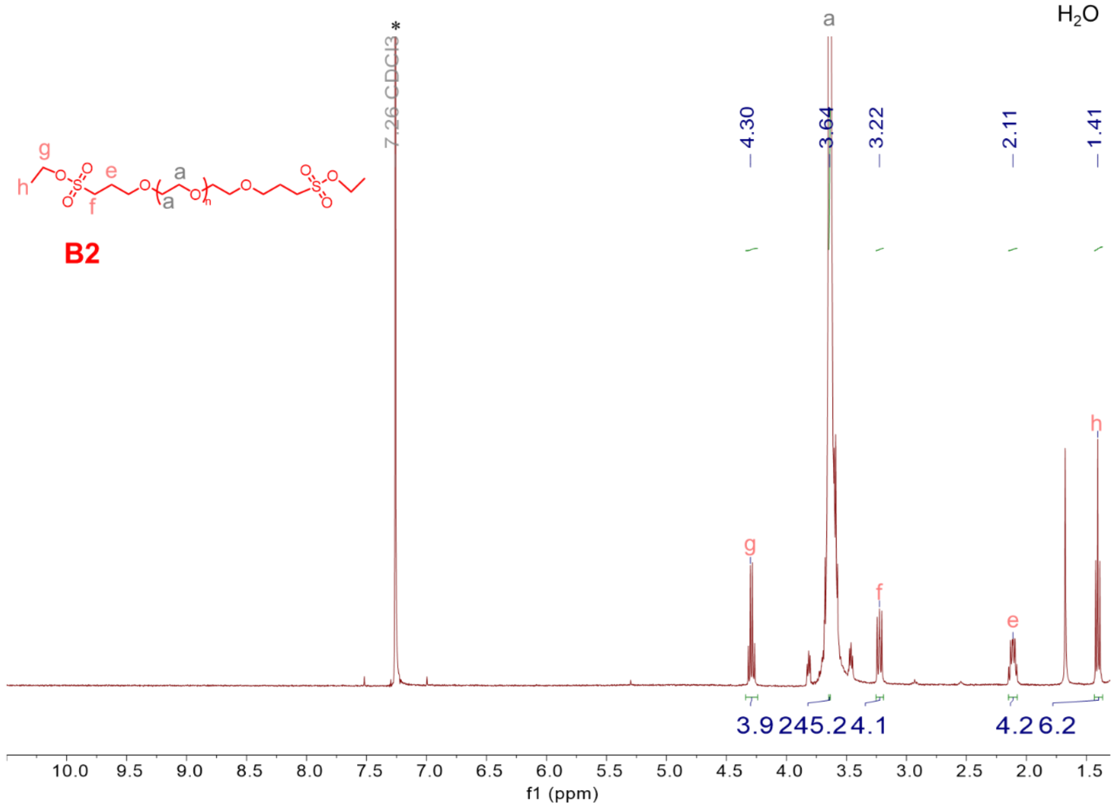

Figure S7. ^{1}H NMR spectrum (in $CDCl_3$[*]) of α,ω-sulfonate ester PEG (B2) prepared by a new synthesis route under neutral conditions, demonstrating nearly quantitative chain-end fidelity.

- **^{1}H NMR data for A2/B2 blend**

(a) Non-stochiometric blending

Figure S8 shows ^{1}H NMR spectrum of a counterion-free A2/B2 blend prepared with an initial molar ratio of 1.4 eq A2 to 0.6 eq B2, i.e., excess imidazole groups are anticipated to remain after blending. Consequently, the spectroscopy should exhibit signals corresponding to 0.6 eq of α,ω-ethyl imidazolium PEG (A2 cation), 0.6 eq of α,ω-sulfonate PEG (B2 anion), 0.8 eq of α,ω-imidazole PEG (neutral A2) and the absence of α,ω-sulfonate ester PEG (neutral B2). The resonance positions for the unreacted neutral A2 (peaks c, d1 and d2) and B2 (peaks e, f, g and h) are demonstrated in Figure S3 and S7, respectively. The *in-situ* ionization proceeds *via* ethyl group transfers from the ethyl sulfonate ester end-groups to the imidazole end-groups, forming ethyl imidazolium (A2 cation)/sulfonate (B2 anion) ion pairs.

Significant shifts were observed for the methylene protons adjacent to the end groups upon *in-situ* ionization. The methylene signal adjacent to imidazole group shifted downfield from peak c (δ_H 4.11 ppm) to c' (δ_H 4.56 ppm) upon conversion to the imidazolium cation, consistent with increased deshielding due to the positive charge.[1, 2] Meanwhile, the methylene protons near sulfonate shifted upfield from peak f (δ_H 3.23 ppm) to f'(δ_H 2.92 ppm), reflecting the transition from a neutral ester to a negatively charged sulfonate anion.[3, 4] These spectral changes confirm the successful formation of imidazolium-sulfonate ion pairs. For the ethyl group protons, following ethyl groups transfer from the ethyl sulfonate ester end-groups to the imidazole end-groups, the methylene protons of the ethyl groups remain unchanged (peaks g and g' both at δ_H 4.31 ± 0.01 ppm), while the methyl protons of ethyl groups shift downfield from peak h (δ_H 1.41 ppm) to h' (δ_H 1.58 ppm). After *in-situ* ionization, the imidazole proton resonances (peaks d1 and d2) shift downfield to those characteristic of the imidazolium cation (peaks d1' at δ_H 10.12 ppm, and d2' at 7.63 ppm and 7.23 ppm).

Using the integral of the repeating ethylene glycol units (peak a) as a reference (245.2), the integrals of other peaks were analyzed to confirm the presence of the expected species. The 0.6 eq of α,ω-ethyl imidazolium PEG (A2 cation) and 0.6 eq of α,ω-sulfonate PEG (B2 anion) were confirmed by the observed integrals of peak d1' (0.6, expect 0.6), d2' (0.6 + 0.7 = 1.3, expected 1.2), c' (1.1, expected 1.2) and f' (1.2, expected 1.2). The presence of 0.8 eq of α,ω-imidazole PEG (neutral A2) was confirmed the observed integrals of peak c (1.5, expect 1.6), d1 (0.8, expected 0.8) and d2 (1.5, expected 1.6). Finally, the complete reaction of B2 (the complete disappearance of the α,ω-sulfonate ester PEG, neutral B2) was confirmed by the disappearance of peaks f (δ_H 3.23 ppm) and h (δ_H 1.41 ppm).

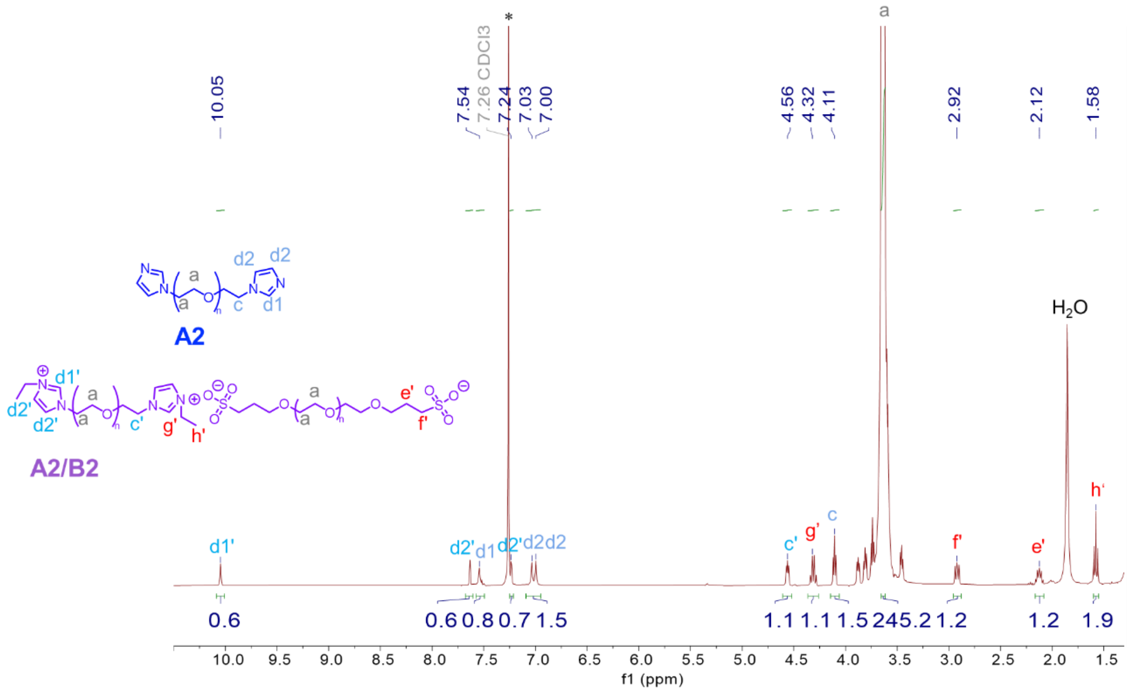

Figure S8. ^{1}H NMR spectrum (in $CDCl_3$) of a counterion-free A2/B2 blend prepared with an initial molar ratio of A2:B2=1.4:0.6 (B2 fully reacted).*

(b) Stochiometric blending

Figure S9 shows ^{1}H NMR spectroscopy of a counterion-free A2/B2 blend prepared at a 1:1 molar ratio. Near-quantitative conversion to ion pairs was evidenced by the near-complete disappearance of the characteristic imidazole protons resonances (peaks d1 at δ_H 7.53 ppm, and d2 at 7.04 ppm and 7.00 ppm, as observed in Figures S3 and S8) in the NMR spectrum. Upon *in-situ* ionization, the major set of new resonances characteristic of the imidazolium cation appeared downfield at similar chemical shift positions to those observed in Figure S8: d1' (δ_H 10.07 ppm, cf.10.12 ppm in Figure S8) with an integral of 0.8, and d2' (δ_H 7.63 ppm and 7.24 ppm, cf. 7.63 and 7.23 ppm) with a combined integral of 1.8 (0.9 + 0.9). Additionally, a minor set of imidazolium peaks was observed at d1'' (δ_H 9.73 ppm, integral 0.1) and d2'' (δ_H 8.13 ppm and 8.09 ppm, combined integral 0.2).

We hypothesize that the presence of these two sets of imidazolium peaks (d' and d'') arises from the existence of two distinct imidazolium environments, where potentially localized restricted exchange dynamic slows the exchange process to the NMR timescale. The chemical shift positions of d1'' and d2'' are significantly distinct from those of the unreacted imidazole protons observed in Figure S3 and S8, confirming they do not represent residual neutral A2. Integration of these imidazolium signals suggests a distribution of approximately 80−90% in a dominate, likely ion-paired environment (peaks d1' and d2') and ~10−20% in a minor, potentially more "free" imidazolium environment (peaks d1'' and d2'').[5-7]

Further confirming the near-quantitative conversion, the methylene protons adjacent to the neutral end groups in the precursors exhibited characteristic shifts upon ionization. Specifically, the methylene signal adjacent to the imidazole group (peak c) shifts downfield to c' (δ_H 4.57 ppm) after conversion to imidazolium, while the methylene protons adjacent to the sulfonate ester group (peak f) shifts upfield to f' (δ_H 2.93 ppm) upon forming the sulfonate anion. Additionally, the methyl protons of ethyl groups shift downfield to h' (δ_H 1.58 ppm). All these shifts consistent with the observation in Figure S8.

Using the integral of the repeating ethylene glycol units (peak a) as a reference (245.2), the integrals of other peaks were analyzed to further confirm the near-quantitative conversion and the equivalent molar ratio between ionic A2 and B2 species. The combined integrals of the imidazolium protons (d' + d'': 0.8 + 0.1 + 0.2 + 0.8 + 0.9 = 2.8, expected 3) were consistent with the expected stoichiometry. Similarly, the integrals of the methylene protons adjacent to the imidazolium (c': 1.9, expected 2), the methylene protons adjacent to the sulfonate (f': 2.0, expected 2.0), and the methylene protons of the ethyl group (g': 2.1, expected 2) aligned with the expected values. The integrals of the methyl protons of the ethyl group (h': 3.3, expected 3.0) and the methylene protons beta to the sulfonate (e': 2.2, expected 2.0) also supported the near-quantitative conversion.

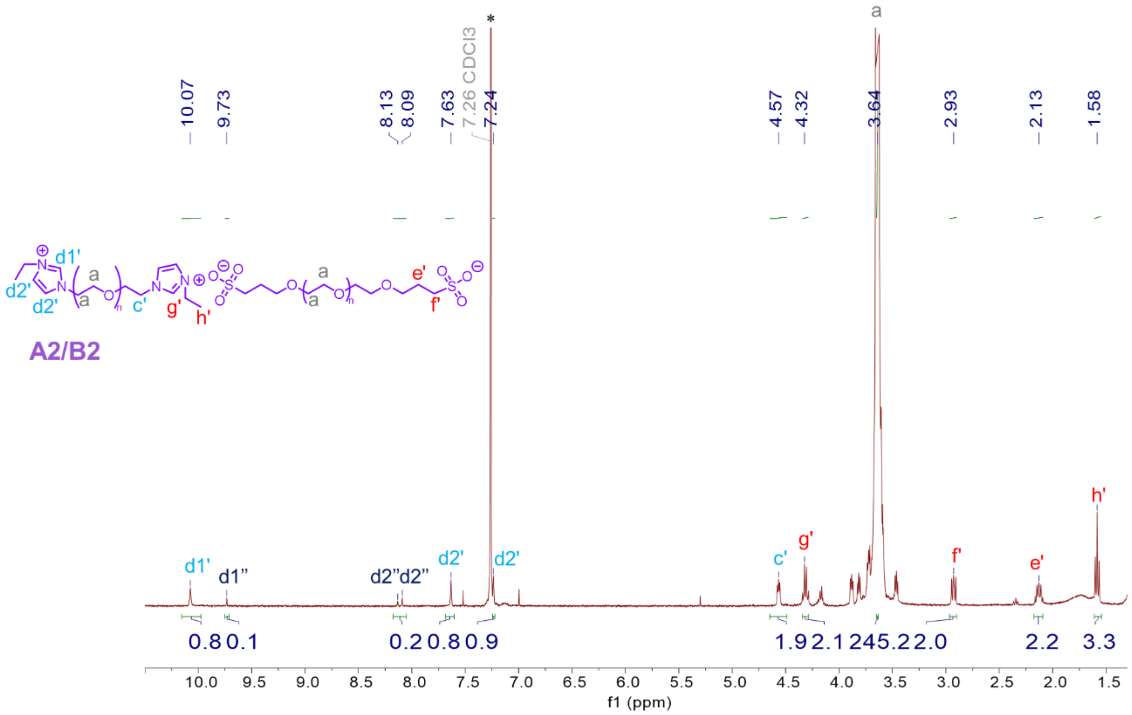

Figure S9. ^{1}H NMR spectrum (in $CDCl_3$*) of a counterion-free A2/B2 blend at a 1:1 molar ratio (fully reacted).

(2) Fourier transform infrared (FTIR) spectroscopy

FTIR spectra were recorded on a Nicolet iS5 FTIR equipped with an iD7 Attenuated Total Reflectance (ATR) accessory. For each measurement, dried bulk polymer powder was compressed against the diamond crystal using an anvil. Spectra were collected at a resolution of 0.974 cm^{-1} and taken from the sum of 50 scans. All absorbance spectra were normalized to the intensity of the C−O stretching peak at ~1100 cm^{-1}.

The *in-situ* ionization between A2 and B2 was monitored by FTIR spectroscopy (Figure S10). In the spectrum of A2/B2, the characteristic peak for the imidazole group of neutral precursor A2 at ~1510 cm^{-1} (C=N and N=C−N stretching)[8] was absent. Similarly, the characteristic peaks for the sulfonate ester in neutral precursor B2 (O=S=O at ~1262 cm^{-1}; S−O−R at ~1006 cm^{-1}, 921 cm^{-1}, and 804 cm^{-1})[9, 10] also disappeared. Concurrently, new absorption peaks emerged in the A2/B2

spectrum, corresponding to the imidazolium cation at ~1562 cm^{-1} (C=N and N=C−N stretching)[11] and sulfonate anion at ~1207 cm^{-1} and 1037 cm^{-1} (S−O stretching)[12]. The complete disappearance of neutral A2 and B2 precursors signals alongside the appearance of these new ionic peaks confirms a near-quantitative ionization.

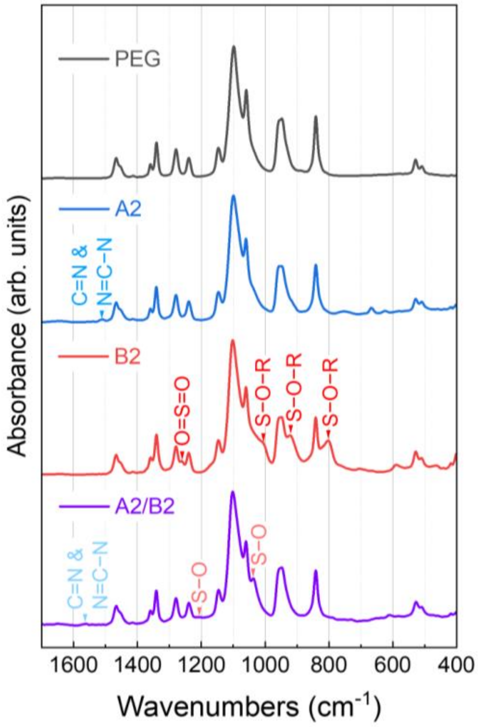

Figure S10. FTIR spectra of PEG, A2, B2 and A2/B2 blend.

(3) Matrix-assisted laser desorption/ionization time of flight (MALDI-TOF) mass spectroscopy

Matrix-assisted laser desorption/ionization time of flight (MALDI-TOF) experiments were performed using a Bruker Microflex MALDI-TOF mass spectrometer (Bruker Daltonics) in optimized conditions in positive linear mode. About 1 mg of sample was dissolved in 200 μL of tetrahydrofuran. Trans-2-[3-(4-tert-Butyl-phenyl)-2-methyl-2-propenylidene] malononitrile (DCTB) (10 mg/mL) and sodium trifluoroacetic acid (NaTFA) (10 mg/mL) were used as a matrix

and cationization agent, respectively. One microliter of sample, 1 µL of NaTFA, and 20 µl of DCTB were mixed thoroughly. About 0.5 µl of this mixture was deposited on a stainless-steel sample holder. After air-drying, the sample was analyzed using MALDI TOF MS. The results of molecular weight characteristics are presented in Table S1, including number average molecular weight (M_n), weight average molecular weight (M_w) and dispersity ($Đ$).

Table S1. Molecular weight characteristics of diol PEG precursor and functional PEG via MALDI-TOF

	M_n (kg/mol)	M_w (kg/mol)	$Đ = M_w/M_n$
PEG	2.88	2.92	1.01
A2	3.05	3.07	1.01
B2	3.17	3.19	1.01

(4) Small-amplitude oscillatory shear (SAOS) tests

Rheological measurements were conducted using a TA Instruments AR-G2 rheometer equipped with an environment test chamber and a 25 mm parallel-plate geometry. An N_2 inert gas purge was employed to prevent air exposure, and a liquid nitrogen (LN_2) cooling system facilitated temperature control. Steady shear experiments were performed at 60 °C for all samples with a shear rate from 1 s^{-1} to 500 s^{-1}. Temperature sweep experiments were conducted using a strain amplitude of 100% and an angular frequency of 6.28 rad/s. The specific temperature ranges and the ramp rates for each sample are shown in Table S2. Dynamic shear experiments were performed at several temperatures to obtain time-temperature superposition (tTs) mastercurves. A strain amplitude of 100% was used for all dynamic shear measurements, which was confirmed to be within the linear viscoelastic (LVE) region. The angular frequency ranged from 0.1 rad/s to 100 rad/s. All mastercurves and shift factors were generated through manual data fitting.[13]

Table S2. The temperature ranges and the ramp rates for temperature sweep experiments

	Temperature range (°C)	Ramp rate (°C/min)
PEG	60−100	2
	90−150	5
A2/B2	50−90	2
	80−150	5
IL/PEG	60−100	2
	90−160	5

The VFT equation (S1) was used to model the shift factor α_T:

$$\alpha_T = \frac{\eta(T)}{\eta(T_r)} = \frac{\eta_\infty \exp(\frac{B}{T - T_0})}{\eta_\infty \exp(\frac{B}{T_{ref} - T_0})} = \exp[B(\frac{1}{T - T_0} - \frac{1}{T_{ref} - T_0})] \qquad \text{(S1)}$$

where B is the VTF parameter, T_0 is the Vogel temperature, T_{ref} is the reference temperature and η_∞ is the viscosity at infinitely high temperature. By fixing T_0 = 155 K,[14] and using the B values obtained from the temperature sweep (Table 2), we calculated the VFT predictions for the shift factors. As shown in Figure S11, the VFT predictions exhibit good agreement with the α_T derived from the frequency sweep data, indicating consistency between the frequency and temperature sweep measurements.

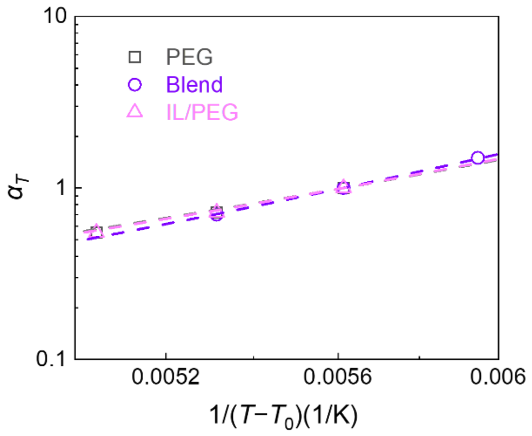

Figure S11. Horizontal shift factor α_T for PEG, A2/B2 and IL/PEG. Dash lines represent VFT prediction using VFT parameters obtained from temperature sweep data (Table 2).

(5) Small- and wide-angle X-ray scattering (SAXS/WAXS)

Small- and wide-angle X-ray scattering (SAXS/WAXS) patterns for the diol PEG precursor and B2 at room temperature were acquired at beamline 11-BM of the National Synchrotron Light Source II (Brookhaven National Laboratory). The SAXS and WAXS patterns were collected using Pilatus2M and Pilatus800k detector systems, respectively. The X-ray beam energy was 13.5 keV, and the sample-to-detector distance were 5 m for SAXS and 0.26 m for WAXS. Measurements were performed at 30 °C. All other SAXS/WAXS data were obtained at Xenocs 3.0 system (Xenocs, Grenoble, France) at the Texas A&M University Soft Matter Facility. This instrument employs a GeniX 3D X-ray beam delivery system to generate monochromatic Cu Kα radiation (λ = 1.54 Å). Scattering patterns were collected using a Dectris Eiger 2R 1M-pixel 2D detector with sample-to-detector distance of 0.900 m for SAXS and 0.045 m for WAXS. Measurements were performed at room temperature and 85 °C. Samples were loaded in 316 stainless steel or aluminum washers and sealed with Kapton tapes. The 2D isotropic scattering patterns were reduced to give a 1D scattering intensity as a function of the wavevector $q = 4\pi \sin(\theta/2)/\lambda$, where θ is the scattering angle and λ is the wavelength.

Small-angle X-ray scattering (SAXS), presented in Figure S12(a), reveals a lamellar structure at room temperature for all samples. SAXS patterns of both diol PEG precursor and IL/PEG exhiibt four Bragg peaks occurring at q^*, 2 q^*, 3 q^*, and 4 q^*, indicating a well-ordered lamellar morphology. In contrast, A2, B2 and A2/B2 blend show only two Bragg peaks at $q^*$and 2 q^*. This suggests that after functionalization, the long-range periodicity decreased, while the presence of the IL salt had minimal influence on this periodicity. The calculated domain spacing ($d = 2\pi/q^*$) for the diol PEG precursor, A2, B2, A2/B2 blend and IL/PEG are approximately 18.5, 10.6, 10.6, 11.9, and 21.7 nm, respectively. Notably, no nanostructure is observed in the melt states from SAXS. Furthermore, wide-angle X-ray scattering (WAXS), as shown in Figure S12(b), indicates that all samples have similar crystalline peaks at room temperature. This peaks completely disappeared in the melt state, confirming that the samples become fully amorphous. The collectively indicates that the chain ends exert no significant influence on the overall crystalline structure of PEG.

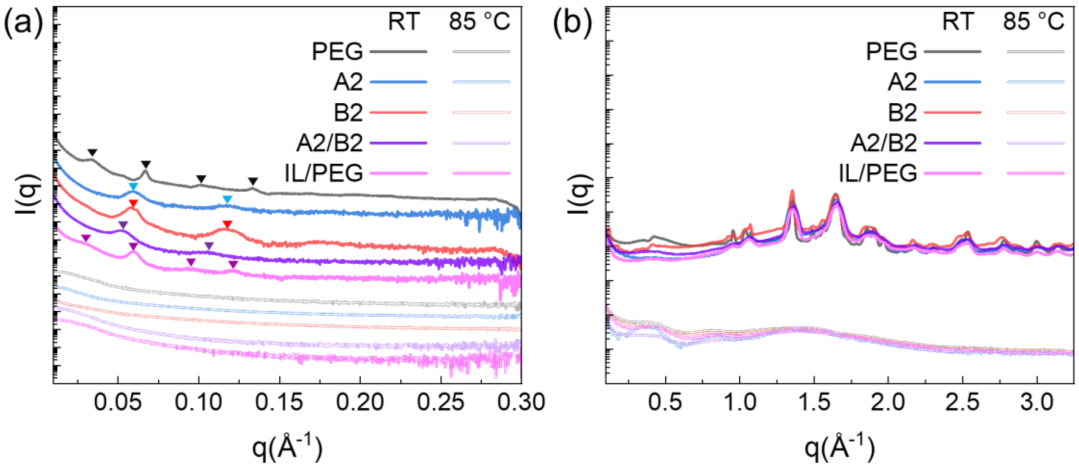

Figure S12. (a) SXAS and (b) WXAS profiles of PEG, A2, B2 and A2/B2 at room temperature and 85 °C.

(6) Pulsed field gradient (PFG NMR) spectroscopy

PFG-NMR measurements were performed on a 400 MHz (9.4T) Bruker wide bore spectrometer and a DiffBB broadband probe. Measurements were performed at temperatures of 60 °C, 75 °C and 90 °C, following at least 20 minutes of thermal-equilibration. The temperature of the sample within the magnet was controlled by dry air flowing at a rate of 535 L/hr. A pure ethylene glycol standard was used to confirm the sample temperature calibration. Proton (^{1}H) self-diffusion coefficients were measured with a variable gradient strength stimulated-echo pulse bipolar sequence designed to minimize T_2 signal loss and minimize background magnetic field gradients.[15]

Self-diffusion coefficients, D_i, for the respective ions were determined by fitting the measured signal intensity I_i as a function of the variable gradient strength (G), using the Stejskal–Tanner equation:

$$I = I_0 \exp\left(-D_i G^2 \gamma^2 g^2 \delta^2 \left(\Delta - \frac{\delta}{3}\right)\right), \qquad \text{(S2)}$$

where I is the normalized signal intensity, I_0 is the initial signal intensity for a given component, γ is the gyromagnetic ratio, δ is the gradient pulse duration, and Δ is the total diffusion time. For the A2/B2 blends, an additional stretched exponential parameter β demonstrated an improved fit over equation S2. The resulting self-diffusion coefficients obtained from this stretch exponent fit were similar to those from the single exponent fit, differing by less than 3% for all temperatures.

$$I = I_0 \exp\left(-D_i G^2 \gamma^2 g^2 \delta^2 \left(\Delta - \frac{\delta}{3}\right)\right)^{\beta}, \qquad \text{(S3)}$$

A visualization of the stimulated echo pulse sequence is shown in Scheme S1. Gradient strength values, G, as well as δ and Δ were optimized to ensure an adequate decay window to accurately determine self-diffusion.

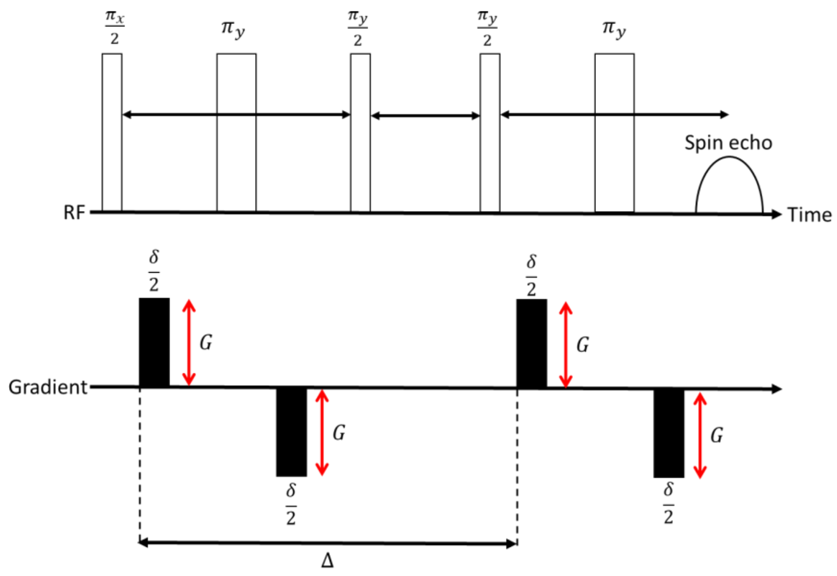

Scheme S1: Basic representation of the Cotts bipolar stimulated-echo pulse sequence employed in PFG-NMR measurements[15]

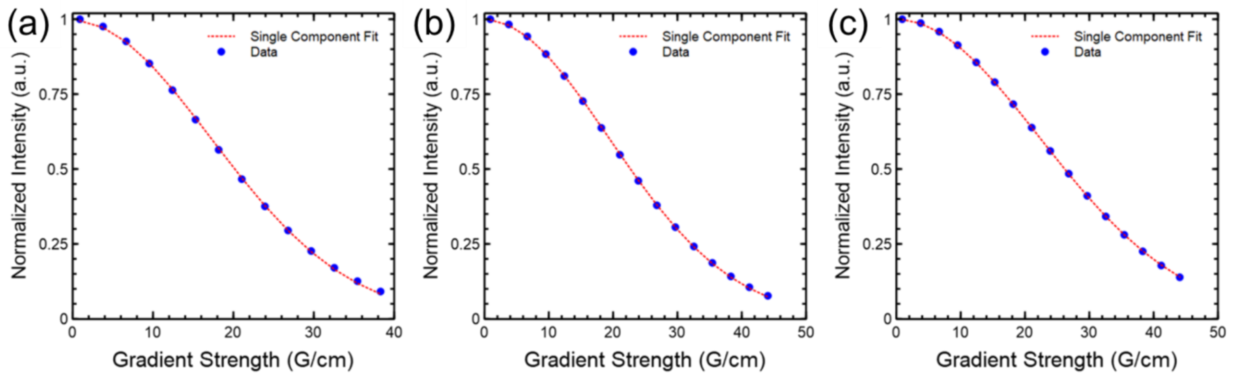

Figure S13. ^{1}*H PFG-NMR decays of the diol PEG polymer at temperatures of (a) 90 °C, (b) 75 °C and (c) 60 °C.*

Table S3. Self-diffusion coefficients for diol PEG at temperatures of 90 °C, 75 °C and 60 °C

Temperature (°C)	D_{PEG} (m^2/s)	+/- (m^2/s)
90	7.50×10^{-12}	1.2×10^{-13}
75	4.90×10^{-12}	7.0×10^{-14}
60	3.20×10^{-12}	2.3×10^{-14}

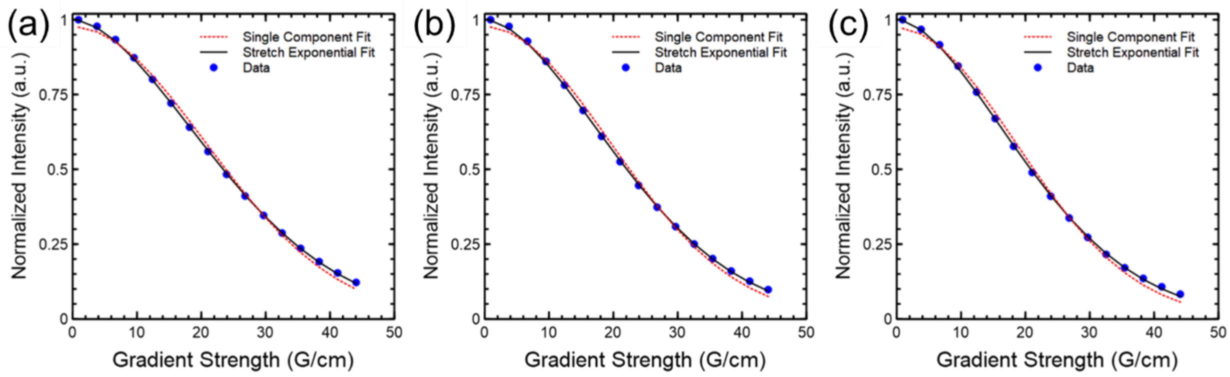

Figure S14 ^{1}H PFG-NMR decays of the A2/B2 blend polymer at temperatures of (a) 90 °C, (b) 75 °C and (c) 60 °C.

Table S4. Single Exponent and Single Stretch Exponential Self-Diffusion Coefficients and Stretch Exponential β for the A2/B2 blend

Temperature (°C)	Single exponent fit		Stretch exponent fit		
	$D_{A2/B2}$ (m²/s)	+/- (m²/s)	$D_{A2/B2}$ (m²/s)	β	+/- (m²/s)
90	4.31×10^{-12}	1×10^{-13}	4.42×10^{-12}	0.89	1.11×10^{-13}
75	2.66×10^{-12}	1.0×10^{-14}	2.73×10^{-12}	0.89	1.0×10^{-14}
60	1.59×10^{-12}	2×10^{-13}	1.65×10^{-12}	0.88	1.9×10^{-13}

(7) Differential scanning calorimetry (DSC)

Melting temperature (T_m) was determined by TA Instruments DSC 2500 or Q200. Polymer samples (3–5 mg) were loaded in Tzero pans and sealed with Tzero lids. Experiments using the DSC 2500 involved two heating and one cooling cycles. For the Q200, two heating and two cooling cycles were performed. A ramp rate of 10 °C/min was used for all heating cycles, and 20 °C/min was used for all cooling cycles.

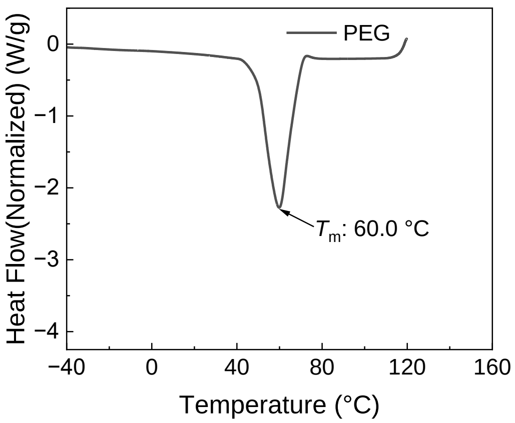

Figure S15. Differential scanning calorimetry spectrum for PEG, indicating a melting temperature (T_m) of 60.0 °C.

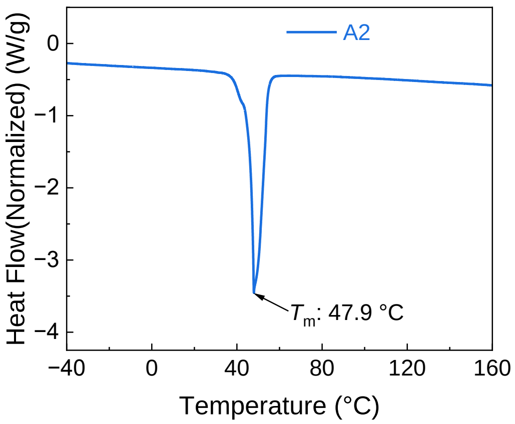

Figure S16. Differential scanning calorimetry spectrum for A2, indicating a melting temperature (T_m) of 47.9 °C.

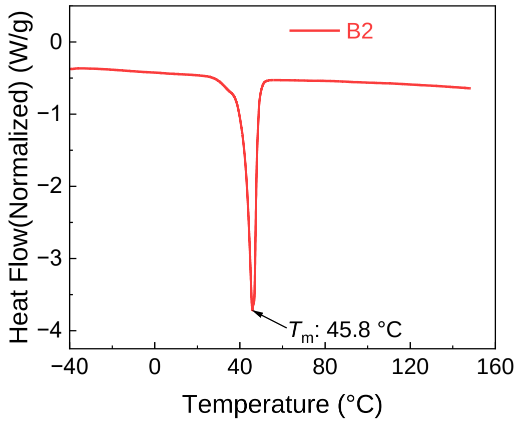

Figure S17. Differential scanning calorimetry spectrum for B2, indicating a melting temperature (T_m) of 45.8 °C.

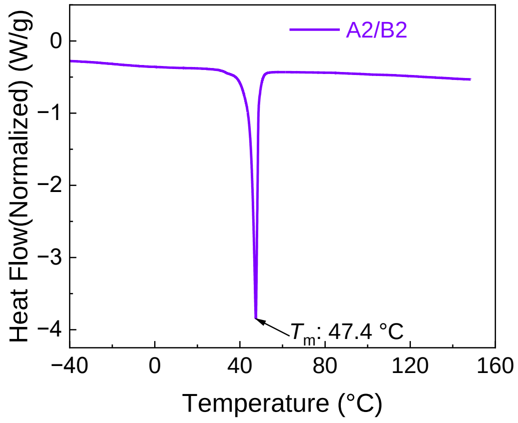

Figure S18. Differential scanning calorimetry spectrum for A2/B2, indicating a melting temperature (T_m) of 47.4 °C.

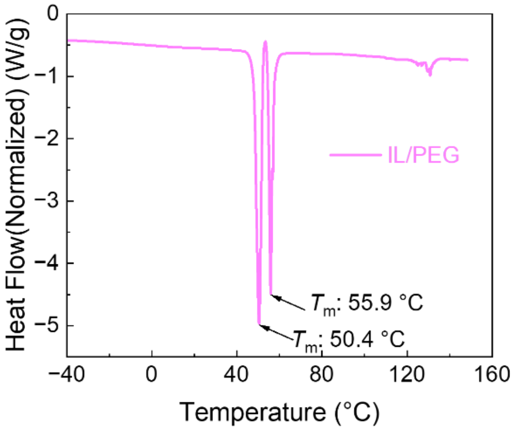

Figure S19. Differential scanning calorimetry spectrum for IL/PEG, indicating melting temperatures (T_m) of 50.4 °C and 55.9 °C.

(8) Thermogravimetric analysis (TGA)

The thermal stability was analyzed using a TA Instrument TGA 5500. Samples of approximately 3−5 mg were heated at 20 °C/min from room temperature to 100 °C followed by a 10-minute isothermal step. After that the samples were heated at 10 °C/min to 600 °C. The whole tests were protected under an N_2 flow of 25 mL/min. The decomposition temperature (T_d) was defined as the temperature corresponding to 5% weight loss from the initial mass.[16]

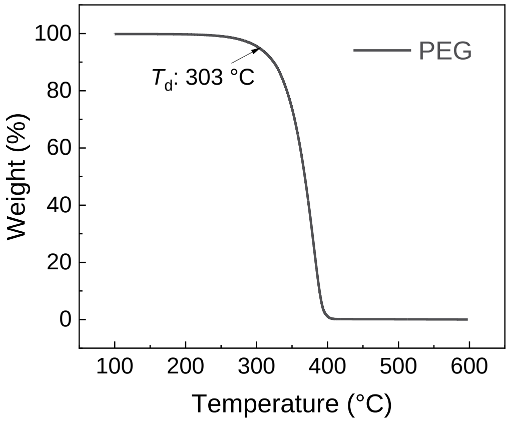

Figure S20. TGA-thermograms of PEG, indicating a decomposition temperature (T_d) of 303 °C.

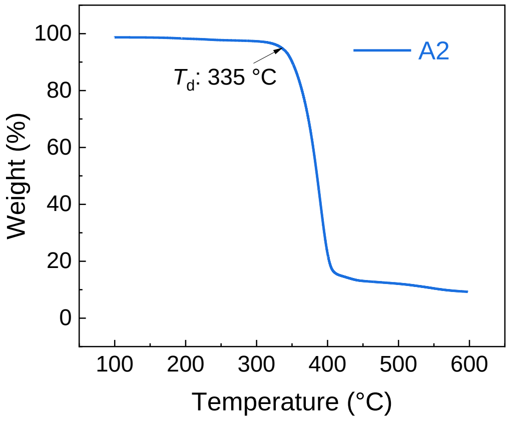

Figure S21. TGA-thermograms of A2, indicating a decomposition temperature (T_d) of 335 °C.

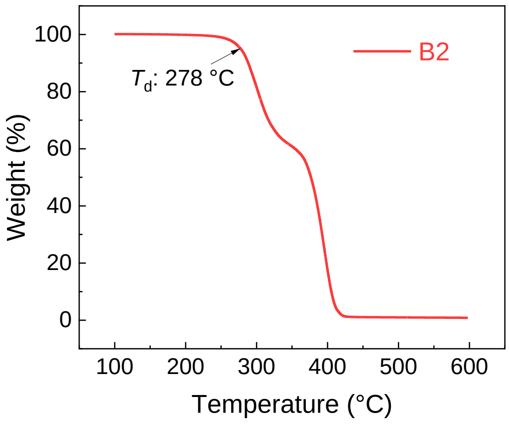

Figure S22. TGA-thermograms of B2, indicating a decomposition temperature (T_d) of 278 °C.

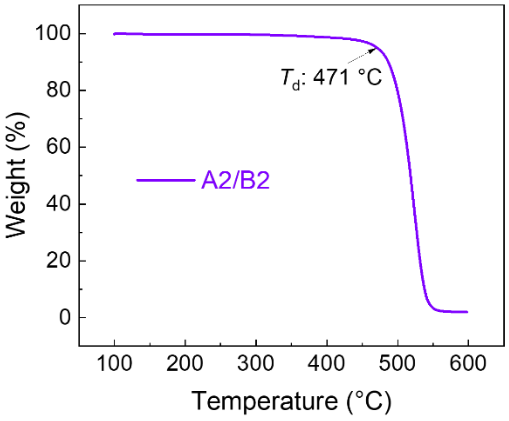

Figure S23. TGA-thermograms of A2/B2, indicating a decomposition temperature (T_d) of 471 °C.

(9) Size exclusion chromatography equipped with multi-angle light scattering (SEC-MALS)

Molecular weight and dispersity of diol PEG precursor were characterized using size exclusion chromatography (SEC) equipped with a UV detector (Agilent), a differential reflective index detector (Agilent) and a DAWN 8 ambient eight-angle light scattering detector (Wyatt Technology). THF was used as the mobile phase. A dilute polymer solution was prepared at a

concentration of 5 mg/mL and filtered through a 0.45 μm PTFE syringe filter prior to SEC-MALS characterization. The pump flow rate was 0.5 mL/min, and the column and RI detector were maintained at 30 °C.

Due to the poor solubility and strong column interactions observed for the functionalized polymers and blend, only α,ω-diol PEG (precursor) was characterized by SEC-MALS under these conditions. The refractive index increment (d*n*/d*c*) of PEG used the value of 0.068 mL/g, which was obtained from a Brice-Phoenix differential refractometer with a sealed cell for high-temperature measurements from the reference.[17, 18]

The results are presented in Table S5 and Figure S24. From the SEC data, the diol PEG exhibited a number average molecular weight (M_n) of 3,060 g/mol, a number average molecular weight (M_w) of 3,160 g/mol and a polydispersity ($Đ$) at 1.03.

Table S5. Properties of diol PEG

Properties	
Number average molecular weight (M_n)	3,060 g/mol
Weight average molecular weight (M_w)	3,160 g/mol
Polydispersity (M_w/M_n)	1.03

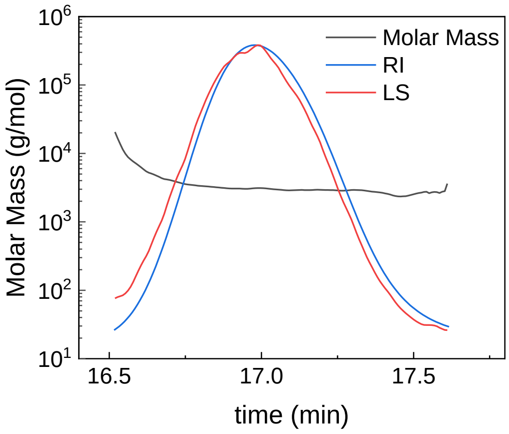

Figure S24. SEC with MALS elugram of diol PEG in THF.

Section C. References

(1) Bara, J. E.; Gabriel, C. J.; Lessmann, S.; Carlisle, T. K.; Finotello, A.; Gin, D. L.; Noble, R. D. Enhanced CO2 Separation Selectivity in Oligo(ethylene glycol) Functionalized Room-Temperature Ionic Liquids. *Industrial & Engineering Chemistry Research* **2007**, *46* (16), 5380-5386.

(2) Yen, W.-P.; Chen, K.-L.; Yeh, M.-Y.; Uramaru, N.; Lin, H.-Y.; Wong, F. F. Investigation of soluble PEG-imidazoles as the thermal latency catalysts for epoxy-phenolic resins. *Journal of the Taiwan Institute of Chemical Engineers* **2016**, *59*, 98-105.

(3) Hofman, A. H.; Pedone, M.; Kamperman, M. Protected Poly(3-sulfopropyl methacrylate) Copolymers: Synthesis, Stability, and Orthogonal Deprotection. *ACS Polym Au* **2022**, *2* (3), 169-180.

(4) Hofman, A. H.; Fokkink, R.; Kamperman, M. A mild and quantitative route towards well-defined strong anionic/hydrophobic diblock copolymers: synthesis and aqueous self-assembly. *Polymer Chemistry* **2019**, *10* (45), 6109-6115.

(5) Chen, S.; Izgorodina, E. I. Prediction of (1)H NMR chemical shifts for clusters of imidazolium-based ionic liquids. *Phys Chem Chem Phys* **2017**, *19* (26), 17411-17425.

(6) Lengvinaite, D.; Klimavicius, V.; Balevicius, V.; Aidas, K. Computational NMR Study of Ion Pairing of 1-Decyl-3-methyl-imidazolium Chloride in Molecular Solvents. *J Phys Chem B* **2020**, *124* (47), 10776-10786.

(7) Du, M. X.; Han, L. X.; Wang, S. R.; Xu, K. J.; Zhu, W. R.; Qiao, X.; Liu, C. Y. Solvent Effects on the (1) H-NMR Chemical Shifts of Imidazolium-Based Ionic Liquids. *Chemphyschem* **2023**, *24* (20), e202300292.

(8) Naumov, P.; Ristova, M.; Šoptrajanov, B.; Zugik, M. Vibrational spectra of bis(acetato)tetrakis(imidazole)copper(II). *Journal of Molecular Structure* **2001**, *598* (2-3), 235-243.

(9) Jin, G.; Jeffrey, M. C.; Douglas, D. A.; Edwards, Q. K. Determination of Sulfate Esterification Levels in Cellulose Nanocrystals by Attenuated Total Reflectance - Fourier Transform Infrared Spectroscopy. In *2010 Pittsburgh, Pennsylvania, June 20 - June 23, 2010*, 2010, 2010; American Society of Agricultural and Biological Engineers: St. Joseph, MI.

(10) Pavia, D. L.; Lampman, G. M.; Kriz, G. S.; Vyvyan, J. R. *Introduction to Spectroscopy*; 2013.

(11) Jheng, L. C.; Hsu, C. Y.; Yeh, H. Y. Anion Exchange Membranes Based on Imidazoline Quaternized Polystyrene Copolymers for Fuel Cell Applications. *Membranes (Basel)* **2021**, *11* (11).

(12) Dereszewska, A.; Olayo, R.; Cardoso, J. Synthesis and thermal degradation of poly(n-butylisocyanate) modified by 1,3-propanesultone. *Journal of Applied Polymer Science* **2003**, *90* (13), 3594-3601.

(13) Ricarte, R. G.; Shanbhag, S. A tutorial review of linear rheology for polymer chemists: basics and best practices for covalent adaptable networks. *Polymer Chemistry* **2024**, *15* (9), 815-846.

(14) Niedzwiedz, K.; Wischnewski, A.; Pyckhout-Hintzen, W.; Allgaier, J.; Richter, D.; Faraone, A. Chain Dynamics and Viscoelastic Properties of Poly(ethylene oxide). *Macromolecules* **2008**, *41* (13), 4866-4872.

(15) Cotts, R. M.; Hoch, M. J. R.; Sun, T.; Markert, J. T. Pulsed field gradient stimulated echo methods for improved NMR diffusion measurements in heterogeneous systems. *Journal of Magnetic Resonance (1969)* **1989**, *83* (2), 252-266.
(16) Diez, S.; Hoefling, A.; Theato, P.; Pauer, W. Mechanical and Electrical Properties of Sulfur-Containing Polymeric Materials Prepared via Inverse Vulcanization. *Polymers (Basel)* **2017**, *9* (2).
(17) Strazielle, P. C. Etude par diffusion de la lumière des hétérogéneités rencontrées dans les solutions de polyoxyéthylène. *Die Makromolekulare Chemie* **2003**, *119* (1), 50-63.
(18) *Polymer Data Handbook: Second Edition*; Oxford University Press, 2009.
